# A unified framework for soft inflatable fabric actuators

**DOI:** 10.1038/s41598-025-25643-8

**Published:** 2025-11-24

**Authors:** Odysseas Simatos, Konstantina Tsintzira, Grigorios M. Chatziathanasiou, Panagiotis Polygerinos

**Affiliations:** Control Systems and Robotics Laboratory (CSRL), School of Mechanical Engineering, Hellenic Mediterranean University, Estavromenos, Heraklion, 71410 Crete Greece

**Keywords:** Soft robotics, Soft inflatable fabric actuators, Soft actuator design, Spring-based framework, Engineering, Mathematics and computing

## Abstract

Soft inflatable fabric actuators are gaining traction in soft robotics due to their lightweight, compliant structures and capacity for generating diverse motions. However, the increasing diversity and structural complexity of their designs present significant challenges for scalable modeling and predictive performance analysis. Here, we present a unified taxonomy of soft inflatable fabric actuators, built around a stiffening actuator as a fundamental unit cell. We introduce a spring-based modeling framework that captures the mechanical behavior of complex actuator types- including elongating, contracting, and bending— through modular combinations of individual units in series or parallel. High-fidelity finite element simulations, validated experimentally, show that the mechanical response of complex multi-chamber actuators can be accurately inferred from the behavior of a single unit. Two case studies demonstrate the framework’s practical utility for task-specific actuator design, eliminating the need for iterative prototyping or computationally expensive modeling. This scalable and generalizable approach enables efficient soft actuator design for a wide range of applications, including but not limited to wearable systems, robotic manipulation, biomedical devices, and adaptive or morphing structures.

## Introduction

Over the past decade, soft robotics has emerged as a transformative area of research, offering compliant, lightweight, and inherently safe alternatives to conventional rigid robotic systems. Central to this progress is the advancement of soft inflatable fabric actuators, which have enabled a wide range of robotic functionalities due to their unique mechanical properties and adaptability^1,2^.

Soft inflatable fabric actuators can display a wide range of deformation modes – including stiffening, contraction, bending, twisting, elongation, and omnidirectional motion^3^ – depending on their geometric configuration and internal architecture. This functional diversity broadens their applicability across numerous domains. Nguyen et al.^3^ presented a comprehensive simulation and design framework for various fabric actuator types tailored for wearable applications. Concurrently, advances in fabrication methods and innovative patterning strategies have expanded their design landscape, facilitating the creation of adaptive or morphing structures with tunable stiffness^4–7^. This growing versatility highlights the increasing integration of soft inflatable fabric actuators in both cutting-edge research and practical robotic systems.

In particular, these actuators have demonstrated significant utility in wearable applications, including soft robotic garments^8–12^ for daily life assistance and rehabilitation^13–18^, addressing both lower^19–22^ and upper limb^15,23–27^ mobility needs. A considerable number of studies have also focused on robotic gloves developed for hand rehabilitation^28,30–33,33^. Beyond wearable systems, soft fabric actuators are being integrated into biomedical devices for applications such as tissue biopsies^34^, endoscopic procedures^35,36^, and artificial heart systems^37^. Additionally, they are also widely integrated into robotic manipulators^38,40–42,42^ and grippers^43,45,45^. Their high force-to-weight ratios and broad deformation capabilities^46–48^ make them ideal for tasks involving heavy object lifting or the manipulation of delicate, irregularly shaped items^48,49^. This ability to combine high output force with compliant, lightweight designs makes them ideal for safe human-robot interaction, particularly in industrial settings^50^. In addition to these applications, fabric-based actuators have also been successfully implemented in aerial robotics, where their lightweight, compliant architecture provides enhanced collision resilience and enables variable stiffness for improved flight control^51^..

**Fig. 1:**
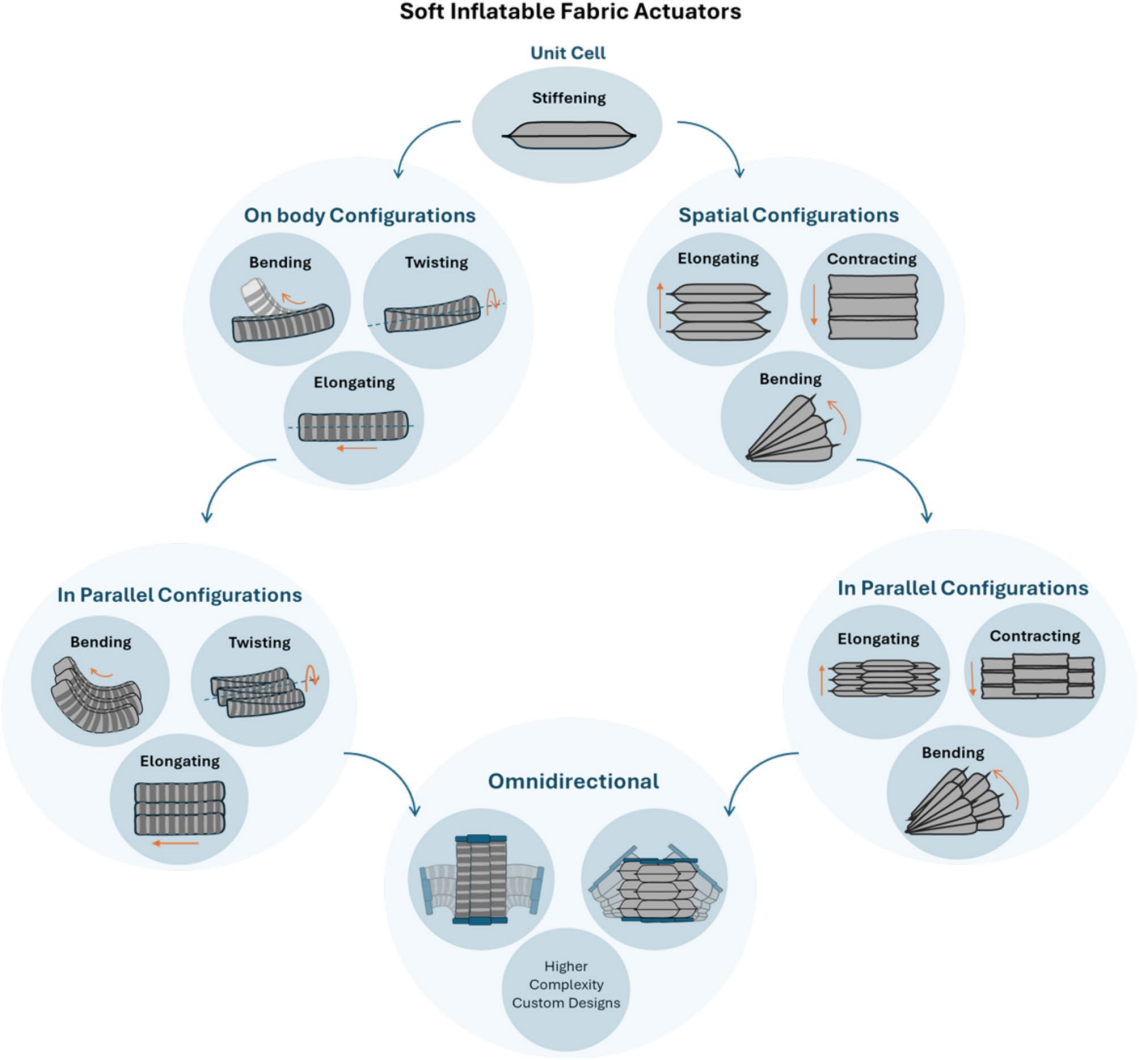
Taxonomy of the soft inflatable fabric actuators based on the fundamental unit cell.

As the field of soft robotics continues to evolve, actuator designs have become increasingly diverse to meet the growing demands of application-specific tasks. Despite this expansion, most research remains focused on the development and evaluation of isolated actuator designs tested under specific experimental conditions. While these studies provide valuable insights into individual actuator performance, their limited scope hinders broader generalization and impedes progress toward scalable and adaptable actuator solutions. Several efforts have aimed to establish predictive models of soft actuator behavior, most notably through analytical modeling approaches developed for pouch motors^48^, and more recently, through data-driven machine learning techniques^52^. However, each method presents critical limitations: analytical models often lack generalizability and fail to capture the complex, nonlinear behavior of soft inflatable systems, while machine learning models – though powerful – typically require extensive training datasets and offer limited physical interpretability.

To advance the generalization of soft actuator performance across diverse applications and design parameters, our previous work^53^ introduced a systematic framework grounded in dimensional analysis. This approach revealed a strong correlation between the performance of a simple unit cell actuator and that of more complex multi-chamber designs. Building upon these findings, the present work introduces a taxonomy of soft inflatable fabric actuators centered around the stiffening actuator, which serves as the fundamental unit cell (Fig. 1). Within this taxonomy, advanced actuator types are conceptualized as spatial configurations composed of multiple unit cells. Additional functionalities arise from on-body modifications of the unit cell, such as embedded reinforcements, which enable complex deformations like twisting, bending, or elongation. Moreover, these spatial and modified configurations can be arranged in parallel to increase force or torque output while preserving individual actuation characteristics. At the highest level of complexity, integrating these enhanced modules supports the design of customized omnidirectional actuators capable of producing motion with multiple degrees of freedom.

Building on this taxonomy, we introduce a structured classification that facilitates a predictive design strategy: the mechanical response of complex actuators can be inferred from the behavior of a single unit cell– the stiffening actuator. This is achieved by modeling the unit cell as a spring element, with more complex actuators represented as assemblies of such elements connected in series or parallel, depending on their spatial configuration. While spring analogies have been previously explored by Yang et al.^54^, primarily for bellows-type actuators, a comprehensive framework unifying all actuator types has not been established until now.

In this study, we extend the spring-based modeling approach to the broader set of spatial and parallel configurations by systematically analyzing the mechanical behavior of the unit cell actuator under various loading conditions, including compression, tension, and bending. High-fidelity finite element method (FEM) models, validated through experimental testing of fabricated prototypes, are used to extract force-displacement or torque-angle relationships. Each deformation mode is mapped to a corresponding spring model (e.g., compression, extension, torsion), enabling accurate prediction of performance in multi-chamber actuator designs. By algebraically combining the responses of individual units, the performance of both spatial and parallel configurations can be predicted, and the need for time-consuming simulations or prototype fabrication for each design is eliminated.

Overall, this work presents a practical and scalable design tool for soft inflatable fabric actuators. By analyzing only the unit cell, accurate performance predictions for complex actuator configurations tailored to specific force or displacement requirements can be rapidly obtained, avoiding computationally intensive simulations and iterative prototyping.

## The stiffening actuator as the unit cell

The stiffening actuator serves as the fundamental unit cell requiring detailed analysis, as its performance characteristics can be used to predict the behavior of more complex actuator configurations. Upon inflation, the actuator undergoes a significant volume increase, resulting in enhanced stiffness and an improved ability to resist external loads. It contracts along both its width and length – with a more pronounced contraction in the width direction – while simultaneously expanding along its height.

**Fig. 2:**
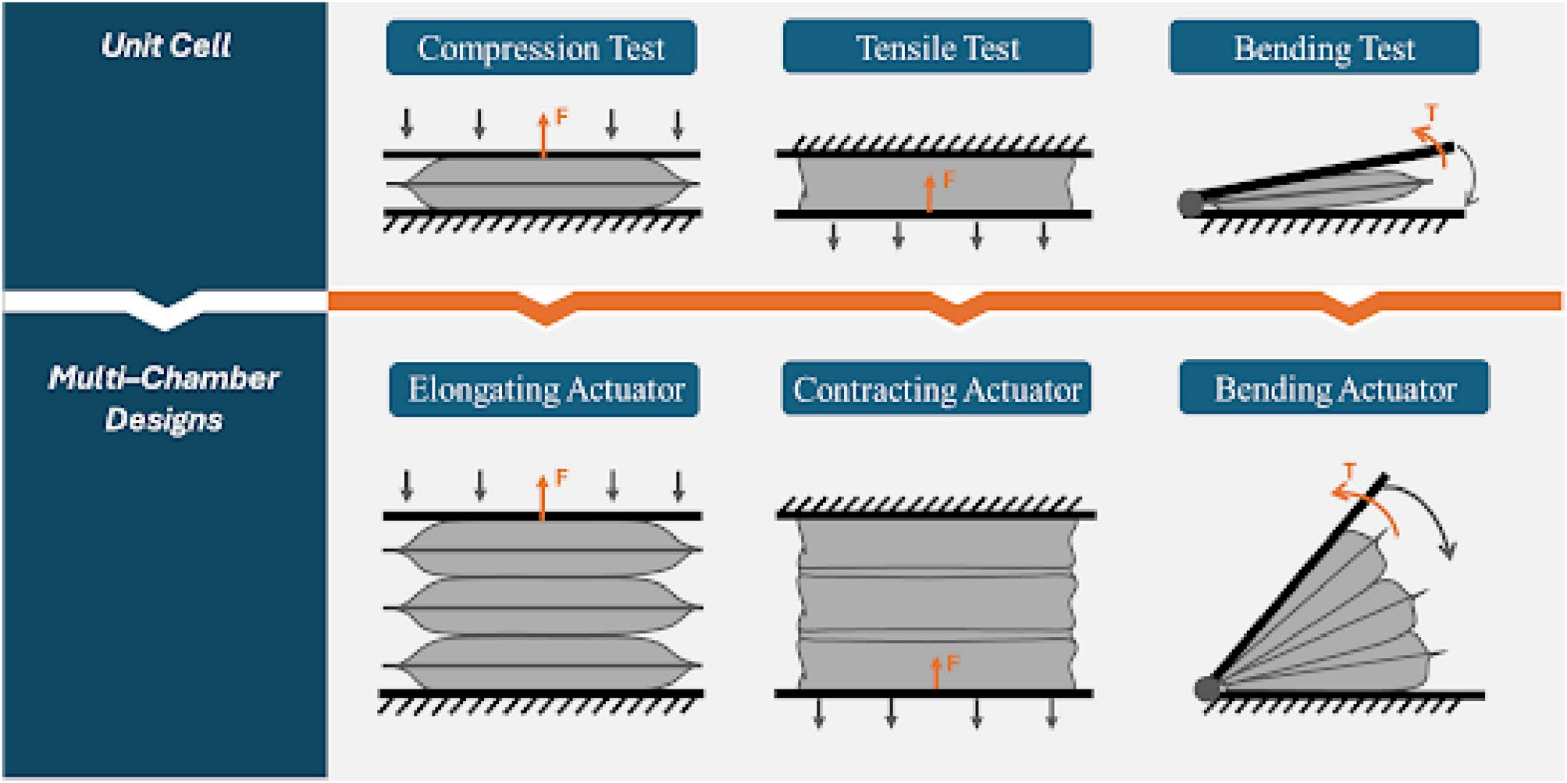
Mechanical testing of the unit cell and the derived multi-chamber designs.

To comprehensively characterize its behavior, three mechanical tests are conducted: compression, tensile, and bending testing. Each test yields performance insights that are transferable to other actuator types. Specifically, compression test results are applicable to elongating actuators, tensile test results relate closely to contracting actuators, and bending test data informs the behavior of bending actuators, as illustrated in Figure 2.

Analyzing the actuator’s performance across these loading conditions requires both extensive experimental testing and computational modeling. To this end, FEM models are employed to simulate and validate the actuator’s response under various experimental conditions. All FEM simulations are conducted using ABAQUS (Simulia, Dassault Systemes). Orthotropic material properties, measured through uniaxial tests conducted according to ISO 527-1, 3 guidelines, are imported into the FEM model. The in-plane elastic moduli are $E_{warp}$ = 361.4 MPa and $E_{weft}$ = 183.3 MPa, the in-plane Poisson ratio is $v_{12}$ = 0.35, and the in-plane shear modulus is $G_{12}$ = 43.2 MPa (see Supplementary Material - Section 1 for more information). Given the thin structure of the fabric layers composing the actuator, S4R shell elements are selected to ensure efficient and accurate simulations. A mesh convergence analysis is performed for all three test cases to determine optimal element size (details provided in the Supplementary Materials - Section 6). The inflation process is represented by a pressure load acting on the inner surfaces of the two layers forming each chamber. A time-dependent ramp gradient, calibrated to the respective experiments, is applied to ensure accurate simulation of the experimental conditions. Tie constraints are applied to simulate the bonded seams, effectively preventing relative motion between fabric layers. The explicit solver is used to accommodate the large, non-linear deformations and rapid inflation characteristics of the soft inflatable actuators. The preprocessing stage of the model—including geometry, material properties, interactions, loads, and mesh—is carefully developed for the unit cell and subsequently extended to multi-chamber actuators using customized Python scripting. All simulations are performed at safe pressure levels, predetermined through experimental testing to ensure the material remains within its linear elastic region.

To validate the FEM models, physical prototypes of the unit cell actuator are fabricated using two layers of TPU-coated nylon fabric (Extremtextil, Germany). The layers are precisely cut using a laser cutter (xTool P2, XTOOL, EU) and sealed along their edges using a heat press (Heat Press 38$\times$38 HP-BASIC, Master Print & Cut Systems, Greece). A Teflon masking sheet is placed between the layers during sealing to prevent bonding in the inflation zones. A pneumatic fitting is mounted on the top layer, allowing connection to an air compressor through a PTFE tube (see Supplementary Materials - Section 2 for further details). The experimental setup is consistent across all mechanical tests, with only minor adjustments specific to each loading condition. The actuator is mounted on a universal testing machine (UTM) (CTM6005, CTM, China) equipped with a 2.5 kN uniaxial load cell. The pneumatic system comprises an air compressor (Compressor K 200–600, AIRPRESS, Germany), a pneumatic controller, and 6 mm tubing. The compressor, which features a 200-liter tank and a maximum pressure of 1.4 MPa, provides a continuous and stable air supply. Pressure control is precisely regulated using a pneumatic regulator integrated with sensors and a microcontroller.

For all mechanical tests – compression, tensile, and bending – the same procedure is followed: an FEM model is developed to simulate the actuator’s response under each loading condition, and the accuracy of the model is validated through experiments using the corresponding fabricated prototypes.

### Mechanical compression of the unit cell

The compression test is designed to evaluate the performance of the unit cell actuator under compressive loads applied along its height. The actuator is initially uninflated and is then pressurized until it reaches full inflation. Following inflation, it is compressed between two rigid plates to a specified displacement, and the resulting reaction force resisting compression is recorded.

**Fig. 3:**
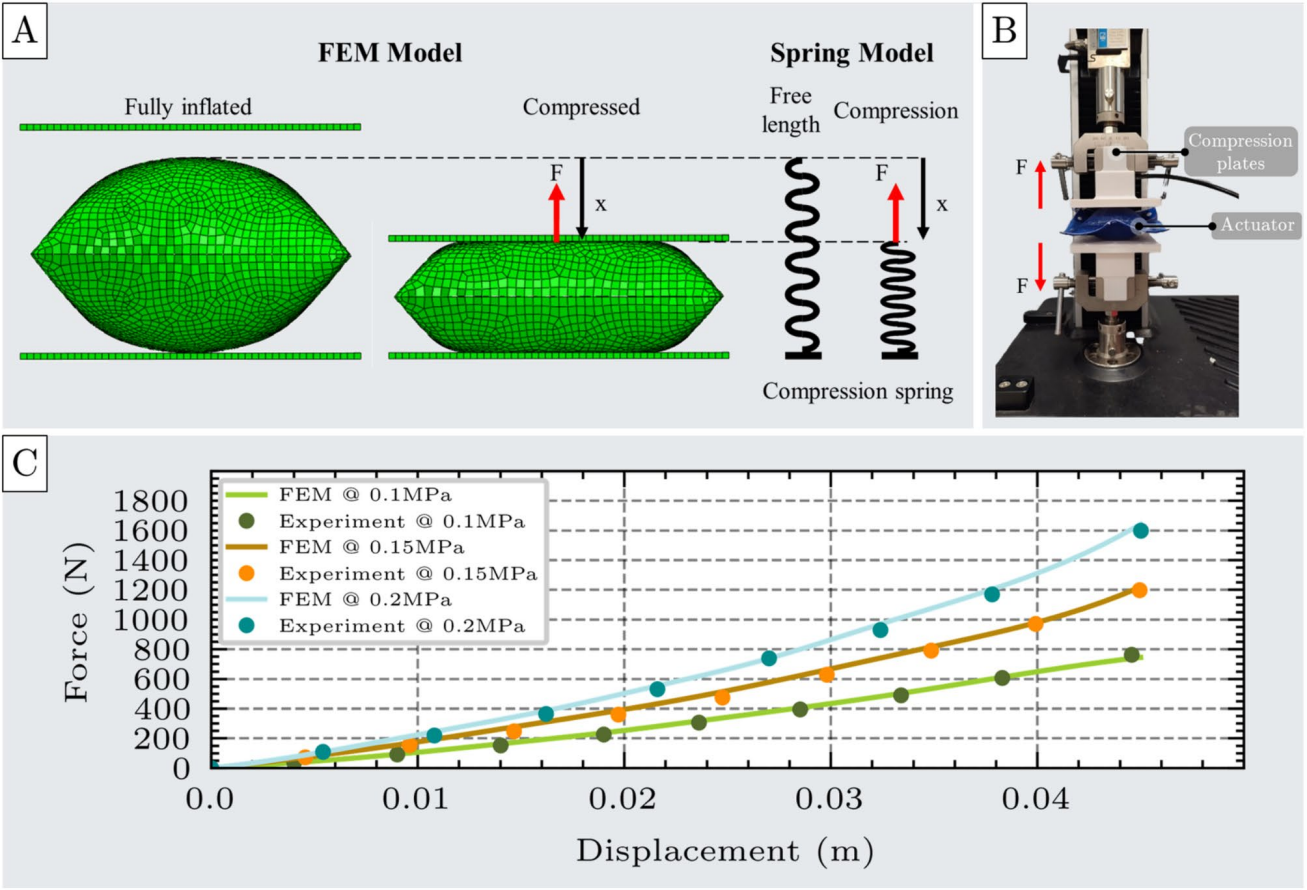
Compression test of the unit cell. (**A**) A Finite Element Method (FEM) model of the unit cell actuator under compression loading, shown alongside an analogous spring model representing the applied force (F) and displacement (x). (**B**) Experimental setup for compression testing, including the actuator prototype, compression plates, and the universal testing machine (UTM). (**C**) Comparison of force-displacement curves obtained from FEM simulations and experimental measurements at three inflation pressures (0.1 MPa, 0.15 MPa, and 0.2 MPa), demonstrating strong agreement between model predictions and experimental data.

To accurately simulate this behavior, the FEM model was implemented in two stages: first, the actuator is inflated to its fully expanded state; second, a prescribed displacement is applied via a moving plate to compress the actuator, while the resulting reaction force was measured, as illustrated in Fig. 3A.

For the experimental validation, a unit cell actuator was fabricated with an uninflated length and width of $l$ = 90 mm and $w$ = 90 mm, respectively, and a material thickness $b$ = 0.6mm (see Supplementary Material - Section 3). In the experimental setup (Fig. 3B), the actuator was secured within a universal testing machine (UTM) and inflated to various pressures at a controlled flow rate of 1.5 SLPM. Once fully inflated, the UTM applied a compressive displacement via its upper crosshead, and the resulting output force (F) was measured using the integrated load cell. This procedure was repeated for multiple displacement values. Each displacement value was experimentally repeated seven times to ensure results are reliable and reproducible. The corresponding mean values and standard deviations are provided in Supplementary Materials, Section 7.

The experimentally measured force outputs and those predicted by the FEM model exhibit strong agreement, with minimal discrepancy, as shown in Fig. 3C. The root mean square error (RMSE) and mean absolute error (MAE) remained consistently low across all three tested pressure levels(RMSE=11.25N, MAE=8.82N). The corresponding force deviations were 3.23$\%$ at 0.1 MPa, 4.92$\%$ at 0.15 MPa, and 3.77$\%$ at 0.2 MPa. These results confirm the accuracy and reliability of the FEM model in predicting the actuator’s behavior under compression.

The actuator exhibited consistent no-load elongation across the tested pressure range of 0.05 to 0.2 MPa. The upper limit of 0.2 MPa was experimentally determined as the maximum safe pressure before the risk of structural failure significantly increases. Much higher-pressure values could be achieved, using emerging fabrication methodologies, such as ultrasonic or radio frequency welding. Substantially higher forces are anticipated at elevated pressure levels, thereby extending the applicability of the actuators to heavy-duty tasks. Conversely, pressures below 0.05 MPa produced overly compliant and insufficiently responsive behavior, falling outside the spring-like regime examined in this study. Within the effective operating range (0.05–0.2 MPa), the actuator demonstrated a stable and repeatable deformation response, with the average total no-load elongation measured at $L_{max}$=44.5 mm.

The results, shown in Fig. 3C for inflation pressures 0.1 MPa, 0.15 MPa, and 0.2 MPa, illustrate the actuator’s force output as a function of displacement. Both the FEM simulations and experimental measurements confirm that the actuator’s resistive force increases with displacement from its free elongation position, exhibiting behavior analogous to a compression spring (Fig. 3A). However, this force-displacement relationship is nonlinear, and a second-order polynomial is required to accurately capture the actuator’s mechanical response. Polynomial fitting was selected as it provides a flexible yet computationally efficient means of approximating nonlinear behaviors while remaining analytically tractable. The polynomial order was determined through regression analyses with increasing degrees, and the lowest-order fit that achieved satisfactory error metrics was adopted to balance accuracy and simplicity. Accordingly, the spring-like behavior is modeled using a quadratic equation, expressed in Eq. 1:

1 $$\begin{aligned} F = k_{e1}(P)\cdot x + k_{e2} (P)\cdot x^2 \end{aligned}$$

where F is the actuator’s force under compression, $k_{e1}$ and $k_{e2}$ are the nonlinear stiffness coefficients of the spring model, respectively, and x represents the displacement from the free elongation position.

**Fig. 4:**
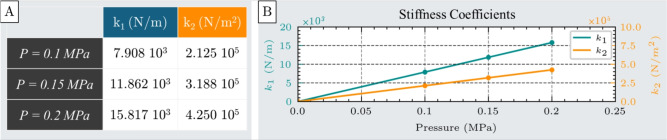
Stiffness coefficients $k_1$ and $k_2$ of the spring model used to characterize the actuator’s nonlinear compression behavior. (**A**) Table of the stiffness coefficients obtained from second-order polynomial fitting of force-displacement data at three different inflation pressures: 0.1 MPa, 0.15 MPa, and 0.2 MPa. (**B**) Pressure-dependent behavior of the stiffness coefficients, showing a linear increase in both terms with inflation pressure.

The stiffness coefficients $k_1$ and $k_2$, obtained through curve fitting at each pressure level, are presented in Fig. 4A. As illustrated in Fig. 4B, both coefficients are pressure-dependent, exhibiting a linear increase with rising internal pressure.

This expression characterizes the mechanical behavior of the unit cell actuator under compression testing across the full range of experimental conditions.

### Mechanical tension of the unit cell

The tensile test is designed to evaluate the performance of the unit cell actuator as it resists tensile loads applied along its width. Initially, the actuator is unpressurized; it is then inflated, resulting in contraction along the width, after which a tensile load is applied to restore it toward its original shape. The force resisting the applied tensile load is then measured.

**Fig. 5:**
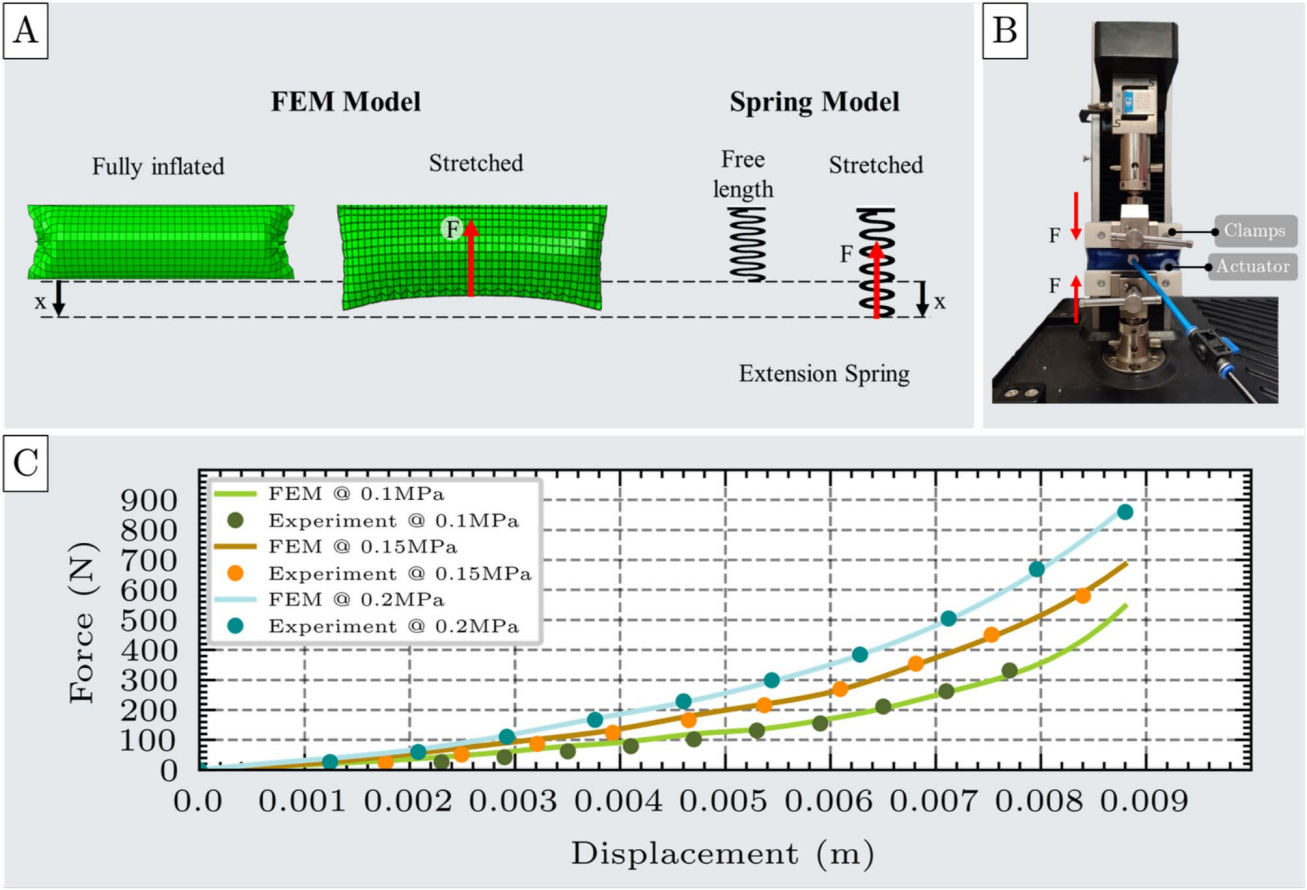
Tensile test of the unit cell. (**A**) FEM model of the unit cell under tensile loading, shown alongside an analogous spring model representing extension behavior with applied force (F) and displacement (x). (**B**) Experimental setup for tensile testing, including the unit cell, custom clamping components, and the UTM. (**C**) Comparison of force-displacement responses from FEM simulations and experimental measurements at three inflation pressures: 0.1 MPa, 0.15 MPa, and 0.2 MPa, demonstrating strong agreement between model and experiment.

The FEM simulation includes three main stages: first, inflating the actuator from its unpressurized to fully inflated state; second, applying a displacement along the lower edge in the direction of the initial width to simulate tensile loading; and third, measuring the resultant force resisting the deformation, as illustrated in Fig. 5A.

For this test, a unit cell actuator with design parameters $l$=100mm, $w$=30mm, $b$=0.6mm was fabricated. In the experimental setup (Fig. 5B), the actuator was mounted on a UTM using custom clamps to prevent slippage. It was then inflated to 0.1 MPa at a controlled flow rate of 1.5 SLPM. The UTM applied incremental displacements while the corresponding resistive force was recorded using the load cell. This process was repeated at higher pressures of 0.15 MPa and 0.2 MPa to assess the actuator’s performance under varying loading conditions. For each pressure level, the experiment was repeated seven times to ensure the results are reliable and reproducible. The corresponding results are shown in Supplementary Materials, Section 7.

The experimental and FEM-derived force outputs showed strong agreement, as illustrated in Fig. 5C. Quantitatively, the RMSE and MAE remained low across all tested pressure levels (RMSE=12.56N, MAE=11.0N), with deviations of 11.03$\%$ at 0.1 MPa, 8.15$\%$ at 0.15 MPa, and 6.64$\%$ at 0.2 MPa. These results confirm the accuracy of the FEM model in simulating the tensile behavior of the unit cell actuator.

Similar to the compression test, the actuator exhibited a nearly constant contracted length across the pressure range of 0.05 to 0.2 MPa. The contracted length of a single chamber stabilized at an average value of $L_{max}$ = 8.8 mm at 0.1 MPa.

Both simulation and experimental results consistently demonstrate that, when the actuator is elongated from its contracted state, it generates a restoring force that increases nonlinearly with displacement, exhibiting behavior similar to that of a nonlinear extension spring (Fig. 5A). This relationship characterizes the actuator’s ability to resist tensile loading and serves as a practical model for predicting its performance during axial extension. Due to the nonlinear nature of this response, the force-displacement relationship cannot be described with a simple linear or quadratic equation. Instead, a fourth-order polynomial is required to accurately represent the mechanical behavior. Accordingly, the spring-like response of the unit cell actuator under tensile loading can be modeled by the equation presented in Eq. 2:

2 $$\begin{aligned} F = k_{c1}(P) x + k_{c2}(P) x^2 + k_{c3}(P) x^3 + k_{c4}(P) x^4 \end{aligned}$$

where F is the actuator’s force resisting tensile deformation, $k_{c1}$, $k_{c2}$, $k_{c3}$, and $k_{c4}$ are the stiffness coefficients of the spring model, and x represents the displacement from the free contracted position.

**Fig. 6:**
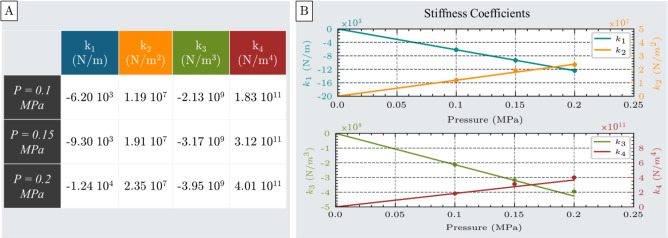
Stiffness coefficients $k_1$, $k_2$, $k_3$, and $k_4$ of the spring model used to characterize the actuator’s nonlinear tensile response. (**A**) Table of stiffness coefficients obtained by fourth-order polynomial fitting of force-displacement data at three different inflation pressures: 0.1 MPa, 0.15 MPa, and 0.2 MPa. (**B**) Linear pressure-dependent behavior of all four stiffness coefficients, indicating a direct correlation between inflation pressure and spring model parameters.

The stiffness coefficients calculated at all pressure levels are shown in Fig. 6A. Similar to the compression test, these coefficients exhibit a linear relationship with pressure, as shown in Fig. 6B.

### Mechanical bending of the unit cell

The bending test evaluates the performance of the unit cell actuator under bending loads. The actuator starts in an uninflated state and is then inflated until fully pressurized. Once pressurized, it is subjected to bending via two rigid, rotating plates that impose a defined angular displacement. In response, the actuator generates a restoring torque that resists the imposed deformation.

**Fig. 7:**
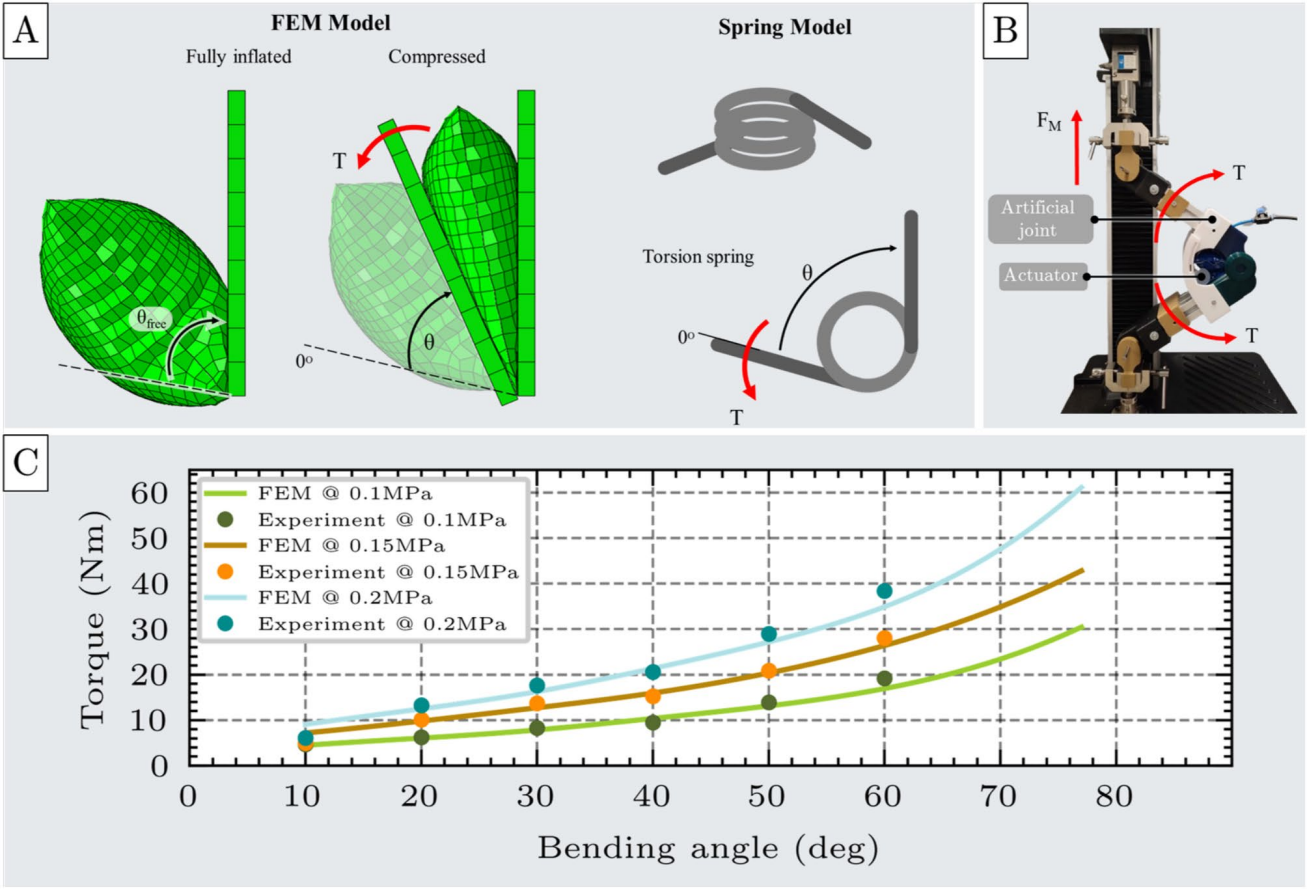
Bending test of the unit cell. (**A**) FEM model of the unit cell under bending load, shown alongside an analogous spring model representing torsional behavior, with applied torque (T) and angular displacement ($\theta$). (**B**) Experimental setup for bending testing, including the unit cell, an artificial joint, and the UTM. (**C**) Comparison of torque-angular displacement responses from FEM simulations and experimental measurements at three inflation pressures: 0.1 MPa, 0.15 MPa, and 0.2 MPa, demonstrating strong agreement between model and experimental results.

The FEM simulation was carried out in two stages: first, the actuator inflated while a semicircular region near one edge remained fixed, indicating a rotational motion; second, an angular displacement was applied via a rotating plate, and the resulting torque generated at the actuator-plate interface was recorded, as illustrated in Fig. 7A.

Experimental validation was performed using a fabricated actuator mounted on an artificial joint attached to the UTM, as shown in Fig. 7B. The actuator was inflated to different pressures at a controlled flow rate of 1.5 SLPM, while the UTM adjusted the joint angle to bend the actuator. At each target angle, the reaction force was recorded using a load cell, and the corresponding torque was calculated (see Supplementary Material - Section 5). This procedure was repeated seven independent times for each experiment across a range of angular displacements (see Supplementary materials - Section 7).

Under no-load conditions, the actuator consistently reached a maximum angular displacement of approximately $80^{\circ }$ across the entire tested pressure range (0.05 to 0.2 MPa).

The results presented in Fig. 7C for 0.1 MPa, 0.15 MPa, 0.2 MPa, illustrate the actuator’s torque output as a function of angular displacement from its free angle $\theta _{free}$. The experimental and FEM simulation results exhibit strong agreement across all pressure levels. Quantitative error metrics remained low, with an RMSE of 1.07Nm and an MAE of 0.81Nm. The corresponding deviations were 6.73$\%$ at 0.1 MPa, 4.61$\%$ at 0.15 MPa, and 3.49$\%$ at 0.2 MPa. These findings validate the accuracy and predictive capability of the FEM model for bending scenarios.

Both simulation and experimental data confirm that the generated torque increases nonlinearly as the actuator bends toward its fully compressed state, exhibiting a restoring response toward the free angle $\theta _{free}$. This behavior is analogous to that of a nonlinear torsional spring. Accordingly, a second-order equation is required to accurately model the actuator’s bending response. The spring-like behavior under bending deformation is described by the expression in Eq. 3:

3 $$\begin{aligned} T = k_{b0}(P) + k_{b1}(P) \theta + k_{b2}(P) \theta ^2 \end{aligned}$$

where T is the actuator torque, $\theta$ is the rotation angle relative to the actuator’s free angle, and $k_{b0}$, $k_{b1}$, and $k_{b2}$ are the stiffness coefficients of the fitted spring model.

The constant term $k_{b0}$ represents the torque exerted at the free angle, arising from the internal bulging of the actuator that occurs even in the absence of external angular displacement (i.e., beyond $\theta _{free}$).

**Fig. 8:**
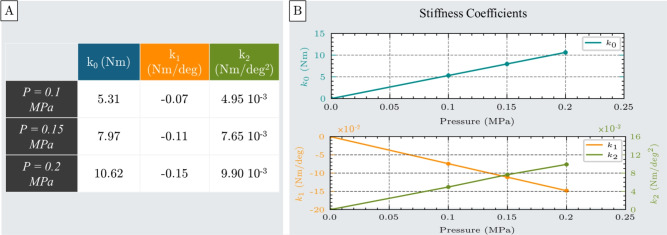
Stiffness coefficients $k_0$, $k_1$, and $k_2$ of the spring model used to characterize the actuator’s nonlinear bending behavior. (**A**) Table of stiffness coefficients obtained from second-order polynomial fitting of torque-angular displacement data at three different inflation pressures: 0.1 MPa, 0.15 MPa, and 0.2 MPa. (**B**) Pressure-dependent behavior of the stiffness coefficients, showing a linear increase in all terms with increasing inflation pressure.

The stiffness coefficients calculated for each pressure level are shown in Fig. 8A. Similar to the compression test, all coefficients exhibit a linear relationship with inflation pressure, as illustrated in Figure 8B.

## Spatial configurations

Multi-chamber actuator designs, such as the elongating, contracting, and bending actuators, can be constructed by spatially combining multiple unit cell actuators. In this section, the behavior of these more complex actuator configurations is approximated by generalizing the mechanical response of individual unit cells under compression, tensile, and bending loads.

For each of these more complex actuator types, high-fidelity FEM models were developed to accurately simulate their mechanical behavior. Corresponding physical prototypes were fabricated and tested to experimentally validate the simulation results.

As illustrated in Fig. 2, the multi-chamber actuators are composed of multiple interconnected unit cells. The previously established spring models for individual actuators are analytically combined to estimate the overall stiffness of the multi-chamber configurations. These analytical predictions are then compared with FEM simulations and experimental measurements to validate the accuracy and applicability of the proposed spring-based modeling approach.

### Elongating actuator

The elongating actuator consists of multiple chambers, each representing an instance of the fundamental unit cell, which was used during the compression test. Following inflation, the actuator extends along the axis, which is perpendicular to the plane of the uninflated actuator, producing elongation. To evaluate its performance, the elongating actuator undergoes the same compression test used to characterize the unit cell actuator.

**Fig. 9:**
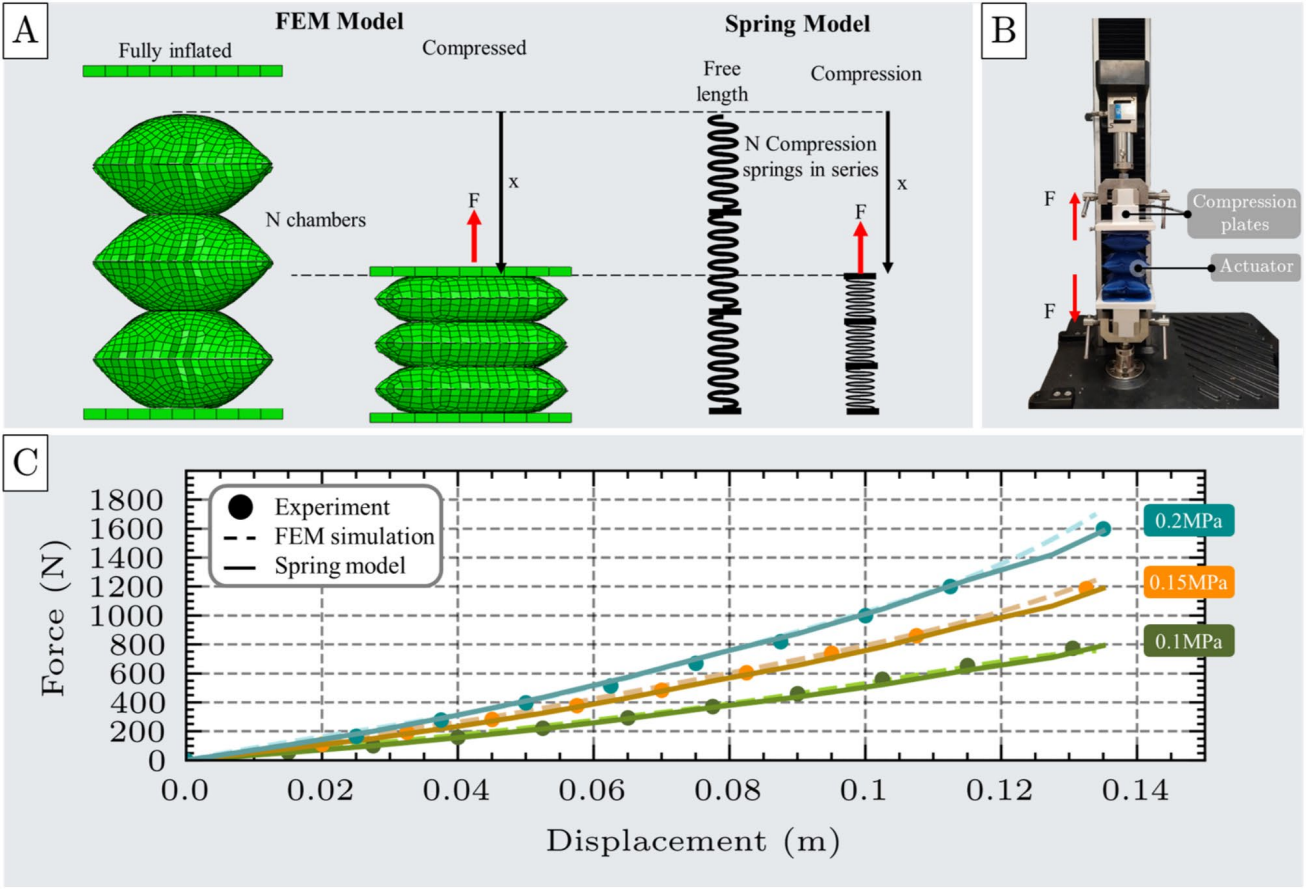
Compression test of the elongating actuator. (**A**) FEM model of the three-chamber elongating actuator under compression loading, shown alongside an analogous spring model comprising three compression springs connected in series, resulting in a uniform force output F under a total displacement x. (**B**) Experimental setup for compression testing of the three-chamber elongating actuator. (**C**) Comparison of force-displacement responses from experimental data, FEM simulations, and the equivalent spring model at three inflation pressures: 0.1 MPa, 0.15 MPa, and 0.2 MPa, demonstrating the predictive accuracy of the simplified spring model.

A FEM model of the elongating actuator was developed using ABAQUS/Explicit (Simulia, Dassault Systèmes), shown in Fig. 9A. The simulation procedure mirrors that of the unit cell’s compression test and includes two stages: first, inflation at rest, and second, compression to a displacement, and measurement of the reaction force opposing the deformation. The experimental setup for the elongating actuator is the same as that used for the compression testing of the unit cell, and it is depicted in Fig. 9B.

The experimental results for the three-chamber elongating actuator demonstrate strong agreement with the FEM simulations across all pressure levels, as shown in Fig. 9C. The RMSE was 14.15N, while the MAE was 10.91N. The overall deviations between FEM predictions and experimental outcomes (5.35$\%$ at 0.1 MPa, 5.48$\%$ at 0.15 MPa, and 4.73$\%$ at 0.2 MPa) confirm that the model reliably captures the mechanical behavior of the elongating actuator.

The behavior of the elongating actuator can be effectively approximated by translating the spring model introduced for the fundamental unit cell under compression (Eq. 1) to multiple equivalent springs in series, as shown in Fig. 9A. In this configuration, each chamber of the elongating actuator, comprising identical unit cell actuators, experiences the same applied force during compression. Consequently, the overall actuator response resembles that of an in-series connection of nonlinear compression springs, where the total displacement is the sum of the individual displacements of each chamber. This modeling approach can be generalized to elongating actuators consisting of N chambers.

To validate the accuracy of the spring model in representing the mechanical behavior of the three-chamber elongating actuator, its predictions were compared against the experimental results across three inflation pressures (0.1, 0.15, and 0.2 MPa). The observed deviations between the spring model and experimental data were 3.53$\%$ at 0.1 MPa, 1.15$\%$ at 0.15 MPa, and 2.75$\%$ at 0.2 MPa. These low error margins confirm that the spring model reliably captures the three-chamber elongating actuator’s compression behavior across a range of pressure levels.

**Fig. 10:**
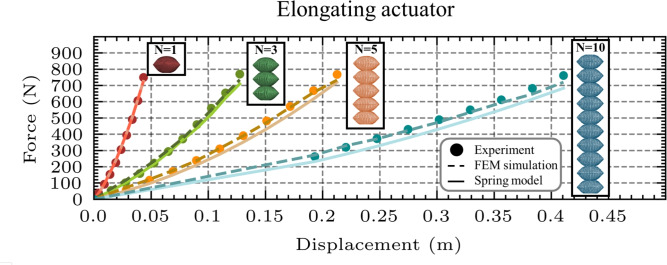
Spring model validation for multi-chamber elongating actuators. Validation of the spring model for elongating actuators with one, three, five, and ten chambers. Across all configurations, the spring-based predictions show strong agreement with both the experimental data and FEM simulation results.

To examine the scalability and validate the spring-based approximation, additional FEM models and physical prototypes were developed for elongating actuators containing five and ten chambers, respectively. Therefore, for an elongating actuator with N chambers, it can be written:

4 $$\begin{aligned}&F = k_{e1}(P) x_1 + k_{e2}(P) x_1^2 = k_{e1}(P) x_2 + k_{e2}(P) x_2^2 = ... = k_{e1}(P) x_N + k_{e2}(P) x_N^2 \end{aligned}$$

5 $$\begin{aligned}&x_{total} = x_1 + x_2 + ... + x_N \end{aligned}$$

where $x_1, x_2, ..., x_N$ are the displacements of each individual chamber from its free end, and $x_{total}$ is the total displacement of the multi-chamber actuator from its free end.

Since all actuators behave identically under the same force, they share the same displacement:

6 $$\begin{aligned} x_{total} = x_1 = x_2 = ... = x_N = \frac{x_{total}}{N} \end{aligned}$$

Substituting, the total applied force, regardless of the number of chambers, can be expressed as a function of the displacement from a free inflated state:

7 $$\begin{aligned} F = \frac{k_{e1}(P)}{N} x_{total} + \frac{k_{e2}(P)}{N^2} x_{total}^2 \end{aligned}$$

This equation calculates the force generated by the actuator when resisting compression, with any number of chambers and any pressure inside the safe pressure limits (0.05–0.2.05.2 MPa). Its validity is demonstrated in Fig. 10, which presents a comparison between the model’s predictions, FEM simulation results, and experimental data for elongating actuators comprising three, five, and ten chambers, all inflated at 0.1 MPa. The observed error levels (3.53$\%$ for the three-chamber actuator, 9.79$\%$ for the five-chamber actuator, and 10.31$\%$ for the ten-chamber actuator) remain relatively low, indicating strong agreement.

The increase in error with a higher number of chambers is attributed to the structural instability observed in multi-chamber actuators. Specifically, beyond five chambers, the actuator tends to exhibit lateral bending upon inflation, deviating from pure vertical extension. To isolate vertical motion, constraints were applied in both simulations and physical experiments to restrict lateral deformation.

Despite this additional complexity in larger actuators, the consistently low error values confirm the reliability of modeling multi-chamber elongating actuators as an in-series configuration of nonlinear compression springs.

### Contracting actuator

The contracting actuator consists of multiple unit cells, connected in such a way that when inflated, the overall length of the actuator is reduced. To evaluate the performance of this actuator type, the actuator undergoes tensile testing: first, the contracting actuator is inflated, next, it is displaced to a specified displacement, and finally, the generated actuator force resisting tensile is measured.

**Fig. 11:**
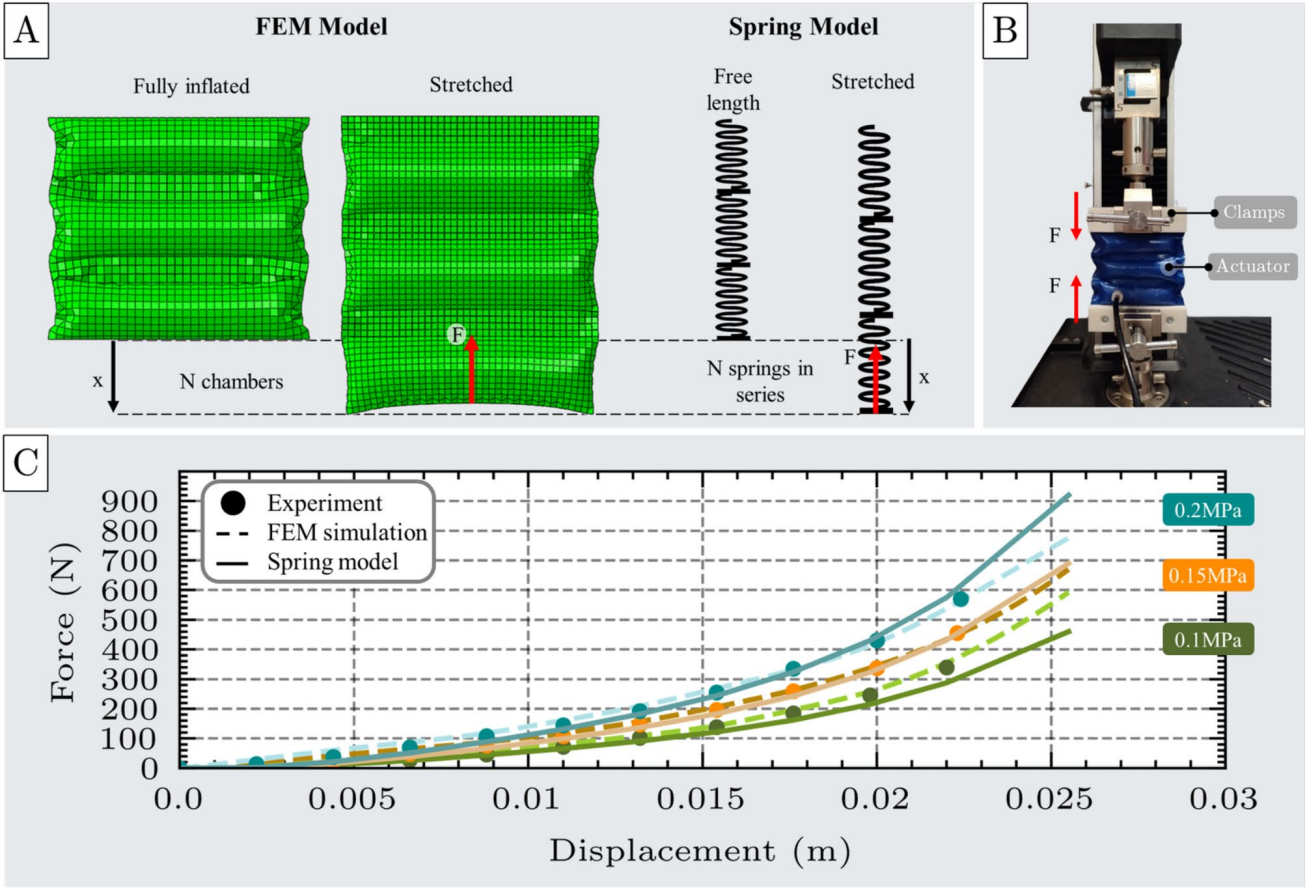
Tensile test of the contracting actuator. (**A**) FEM model of the three-chamber contracting actuator under tensile loading, shown alongside an analogous spring model consisting of three extension springs connected in series, resulting in the same force F, under a total displacement x. (**B**) Experimental setup for tensile testing, including the fabricated three-chamber contracting actuator and the UTM. (**C**) Comparison of force-displacement responses obtained from FEM simulations, physical experiments, and the series spring model at three inflation pressures (0.1 MPa, 0.15 MPa, and 0.2 MPa). Strong agreement across all methods confirms the predictive accuracy of the proposed spring-based model.

The FEM model of the contracting actuator simulating its behavior is developed similarly to the FEM model of the unit cell for tensile testing, and is demonstrated in Fig. 11A. The contracting actuator’s FEM model was developed for a three-chamber contracting actuator to validate its accuracy in simulating the actuator’s behavior. In this study, the contracting actuator was developed with seams of a fixed 10 mm length to ensure consistent results and eliminate any dependence of the outcomes on variations in seam length. The experimental procedure of the contracting actuator, shown in Fig. 11B, followed the same protocol as the tensile test of the unit cell actuator.

The experimentally measured force outputs show strong agreement with the FEM model predictions, as illustrated in Fig. 11C. The two curves follow similar trends, with an RMSE of 9.70N, a MAE of 8.82N, and a deviation of 10.96$\%$ at 0.1 MPa, 8.07$\%$ at 0.15 MPa, and 6.95$\%$ at 0.2 MPa. These low error metrics confirm the accuracy and reliability of the developed FEM model for simulating the contracting actuator’s behavior.

The behavior of the contracting actuator can be effectively approximated by translating the spring model introduced for the fundamental unit cell under tensile testing (Eq. 2) to multiple equivalent springs in series, as shown in Figure 11A. In this configuration, in each chamber of the contracting actuator, the same force is applied during tensile. Consequently, the overall actuator response corresponds to that of an in-series system of nonlinear tensile springs.

To assess the validity of the spring model for the three-chamber contracting actuator, its predictions were compared with experimental results at inflation pressures of 0.1, 0.15, and 0.2 MPa. The deviations between the spring model and the experimental measurements were 11.25$\%$ at 0.1 MPa, 11.39$\%$ at 0.15 MPa, and 11.12$\%$ at 0.2 MPa. These results indicate that the spring-based formulation accurately reflects the actuator’s tensile behavior under varying pressure conditions, confirming its ability to model the three-chamber contracting actuator.

Additional FEM models and physical prototypes of five and ten-chamber actuators were developed to validate this spring-based model approximation for actuators with a different number of chambers. Following the same methodology with the elongating actuator, the contracting actuator’s force as a function of displacement x, pressure P, and number of chambers N can be approximated by the equation below:

8 $$\begin{aligned} F = \frac{k_{c1}(P)}{N} x_{total} + \frac{k_{c2}(P)}{N^2} x_{total}^2 + \frac{k_{c3}(P)}{N^3} x_{total}^3+ \frac{k_{c4}(P)}{N^4} x_{total}^4 \end{aligned}$$

**Fig. 12:**
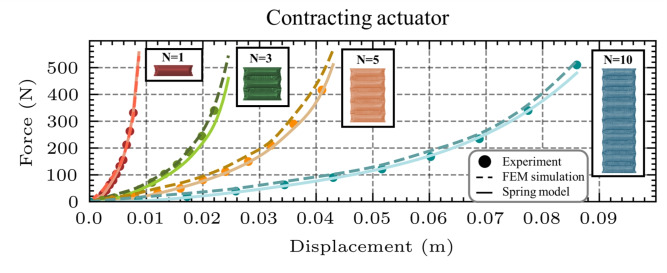
Spring model validation for multi-chamber contracting actuators. Validation of the spring-based model for contracting actuators with one, three, five, and ten chambers. Across all configurations, the spring model demonstrates strong agreement with both experimental measurements and FEM simulation results, confirming its predictive accuracy for multi-chamber systems.

Eq. 8 presents the force generated by the contracting actuator as a function of the number of chambers N and the inflation pressure P within the safe operational range (0.05–0.2MPa). The accuracy of this model is illustrated in Fig. 12, where its predictions are compared against both experimental measurements and FEM simulation results for contracting actuators with three, five, and ten chambers, all inflated at 0.1 MPa. The observed error levels, 11.25$\%$ for the three-chamber actuator, 10.89$\%$ for the five-chamber actuator, and 10.60$\%$ for the ten-chamber actuator, remain acceptably low, demonstrating strong overall agreement. These findings confirm that the spring-based model, originally developed from a single-chamber unit cell, can reliably predict the behavior of multi-chamber contracting actuators.

### Bending actuator

The bending actuator consists of multiple chambers, each representing an instance of the fundamental unit cell. The actuator’s chambers are connected in such a way that upon inflation, the actuator bends and generates a bending torque. To assess its performance, a bending test is conducted, following the same procedure used for the unit cell.

**Fig. 13:**
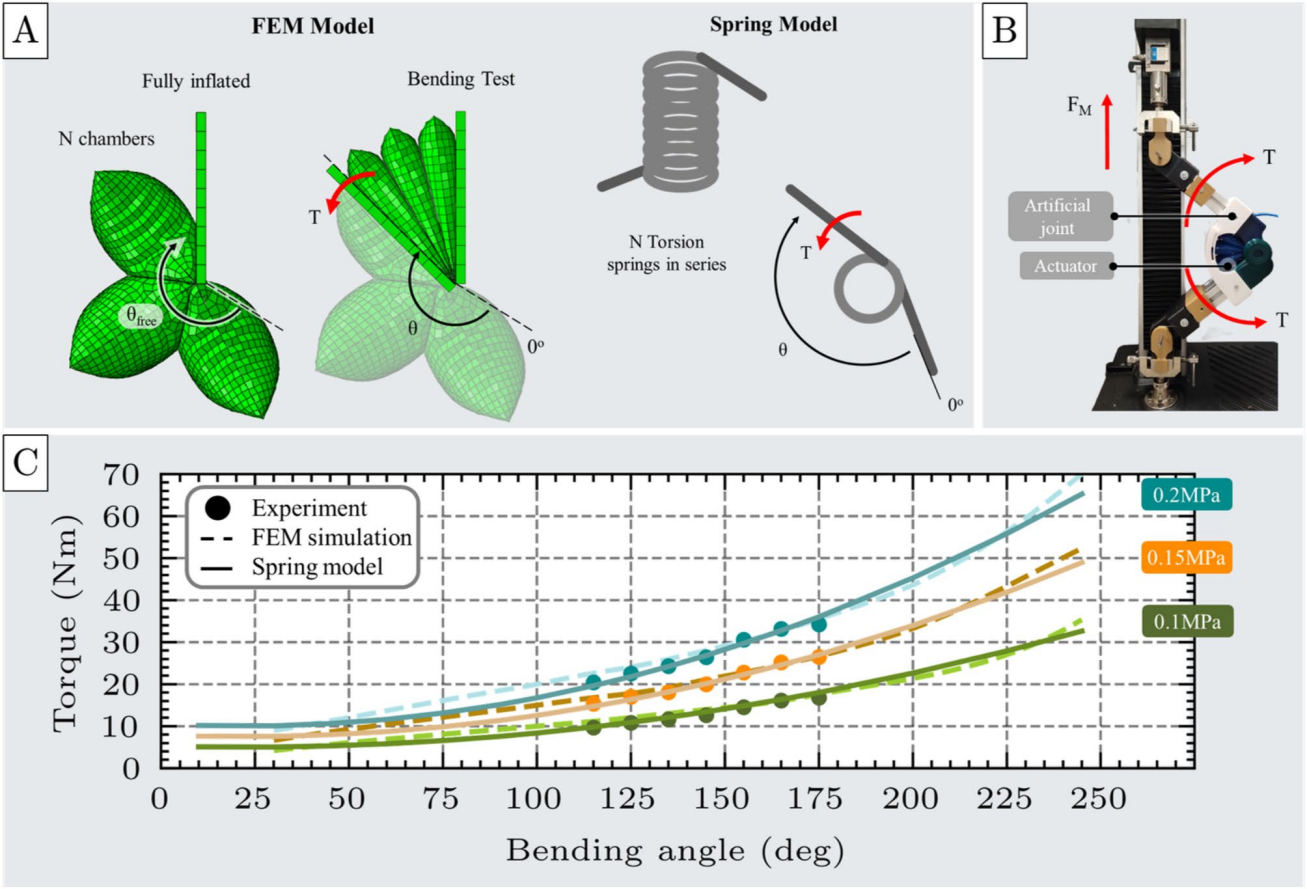
Mechanical test of the bending actuator. (**A**) FEM model of the three-chamber bending actuator under bending loading, shown alongside an analogous spring model comprising three torsional springs connected in series. The model results in the same torque T under a total angular displacement $\theta$. (**B**) Experimental setup used for the bending test of the three-chamber bending actuator. (**C**) Comparison of torque-angle responses from experimental data, FEM simulations, and the spring model at three inflation pressures (0.1 MPa, 0.15 MPa, and 0.2 MPa), demonstrating strong agreement and confirming the predictive reliability of the spring-based model.

A FEM model of the bending actuator was developed using ABAQUS, as shown in Fig. 13A. The simulation procedure mirrors that of the unit cell’s bending test described earlier. As shown in Fig. 13B, the experimental setup for the bending actuator mirrored that used for the bending test of the unit cell actuator.

The experimental results for the three-chamber bending actuator show strong agreement with the FEM simulations across all tested pressure levels, as illustrated in Figure 13C. The RMSE was calculated to be 1.11Nm, and the MAE was 0.95Nm. The overall deviations between the FEM predictions and experimental measurements, 8.07$\%$ at 0.1MPa, 5.15$\%$ at 0.15MPa, and 3.86$\%$ at 0.2MPa, indicate that the model accurately captures the mechanical response of the bending actuator. These results validate the FEM model’s reliability for simulating the behavior of bending actuators.

The behavior of the bending actuator can be effectively approximated by extending the spring model developed for the fundamental unit cell under bending (Eq. 3) to a series arrangement of multiple equivalent torsional springs, as illustrated in Fig. 13A. In this configuration, each chamber of the bending actuator, comprising identical unit cells, experiences the same applied torque during bending. Consequently, the overall actuator response resembles that of a system of nonlinear torsional springs connected in series, where the total angular displacement is the sum of the individual angular deformations of each chamber.

The predictive capability of the torsional spring model was evaluated for a three-chamber bending actuator by comparing its output to the experimental measurements at 0.1, 0.15, and 0.2 MPa. The torque deviations between the model and experimental data were 4.22$\%$, 3.29$\%$, and 2.72$\%$ at each respective pressure level. The close agreement across all cases demonstrates that the torsional spring approximation provides a robust representation of the actuator’s bending response and validates the approach for use in modeling bending actuators.

To further validate this spring-based modeling approach, additional FEM models and corresponding physical prototypes were developed for bending actuators consisting of five and ten chambers. Following the same methodology as with the elongating and contracting actuators, the total torque output of the bending actuator can be expressed as a function of the bending angle $\theta$, pressure P, and the number of chambers N, as shown in the following Eq. 9:

9 $$\begin{aligned} T = k_{b0}(P) + \frac{k_{b1}(P)}{N} \theta _{total} + \frac{k_{b2}(P)}{N^2} \theta _{total}^2 \end{aligned}$$

**Fig. 14:**
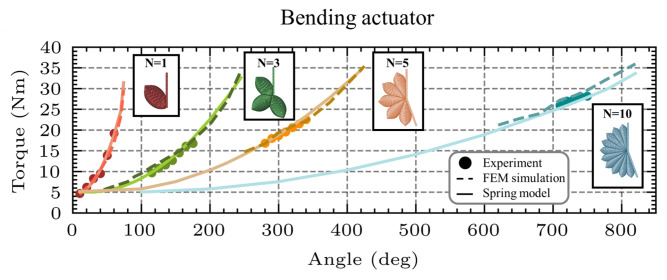
Spring model validation for multi-chamber bending actuators. The proposed spring-based model was validated for bending actuators composed of one, three, five, and ten chambers. Across all configurations, the model exhibited strong agreement with both experimental measurements and FEM simulation results. This consistency confirms the model’s ability to accurately capture the nonlinear torsional behavior of bending actuators, even as the complexity of the system increases with additional chambers.

The proposed spring model (Eq. 9) predicts the torque generated by bending actuators with any number of chambers N at pressures within the safe operational range (0.05–0.2MPa). The validity of this model is illustrated in Fig. 14, where its predictions are compared against both experimental and FEM simulation results for bending actuators with three, five, and ten chambers, when inflated at 0.1MPa. The observed deviations, 4.22$\%$ for the three-chamber actuator, 2.47$\%$ for the five-chamber actuator, and 2.27$\%$ for the ten-chamber actuator, remain relatively low, indicating strong agreement.

The slight decrease in error observed for actuators with a higher number of chambers is due to the limitations of the experimental setup. Actuators with more than four chambers exhibit free bending angles exceeding 360 degrees (a full rotation), preventing both the FEM simulations and experiments from measuring the generated torque beyond this angle. As a result, for actuators with more than four chambers, measurements and simulations are restricted to the portion of their bending range that remains within the measurable domain.

These findings confirm that the spring-based model derived from a single bending unit effectively captures the torque response of multi-chamber bending actuators, supporting its utility as a generalized and scalable performance estimation approach.

In multi-chamber bending actuators with a large number of chambers (5 and 10), their free end exceeds the observable rotation angle of the circle ($360^{\circ }$). However, in the experimental setup, such large rotations cannot be physically realized or measured. As a result, torque measurements in simulation and experiment are only feasible within a limited angular range, significantly below the free rotation angle, where the actuator remains in a more compact and experimentally accessible configuration. Therefore, the torque applied by these bending actuators is always close to the full suppression of each actuator, therefore producing very high torque in all the available angle range, as seen in Figure 14.

## Parallel configurations

In the previous section, it was demonstrated that all types of multi-chamber actuator designs (elongating, contracting, and bending actuators) can be effectively modeled as a system of springs connected in series. This configuration reduced the overall stiffness compared to the fundamental unit cell but allowed for greater displacement, making it suitable for tasks requiring large deformations. However, in many applications, it is essential to increase the actuator’s stiffness and, thus, the corresponding force or torque output for a given displacement. This requirement could be achieved by obtaining actuators that can be modeled as springs connected in parallel.

To achieve this, instead of adding more chambers, multiple actuators of the same type can be arranged in a parallel configuration. In such an arrangement, each actuator unit undergoes the same displacement, and the total force or torque generated by the actuator system during mechanical testing (i.e., compression test for elongating actuators, tensile test for contracting actuators, and bending test for bending actuators) is equal to the sum of the forces or torques respectively, generated by the individual actuators. Accordingly, regarding elongating and contracting actuators, for an actuator system consisting of $N_a$ actuator units, the total output force can be expressed as the sum of the individual forces produced by each unit:

10 $$\begin{aligned} F_{total} = \sum _{i=1}^{N_a} F_i \end{aligned}$$

Similarly, for a bending actuator system consisting of $N_a$ actuator units, the total output torque can be expressed as the sum of the individual torques produced by each unit:

11 $$\begin{aligned} T_{total} = \sum _{i=1}^{N_a} T_i \end{aligned}$$

Specifically, for an actuator system composed of $N_a$ identical elongating actuators, the total output force can be written as:

12 $$\begin{aligned} F_{total} = \left( \frac{k_{e1}(P)}{N} x + \frac{k_{e2}(P)}{N^2} x^2\right) N_a \end{aligned}$$

Equation 12 expresses the total force generated by $N_a$ elongating actuators connected in parallel as a function of their displacement and the number of actuators. Similarly, Equations 13 and 14 extend this formulation to the contracting and bending actuators, respectively, capturing the relationship between actuator number, displacement or angle, and total force or torque output for each actuator type.

13 $$\begin{aligned} F_{total}= & \left( \frac{k_{c1}(P)}{N} x + \frac{k_{c2}(P)}{N^2} x^2 + \frac{k_{c3}(P)}{N^3} x^3+ \frac{k_{c4}(P)}{N^4} x^4\right) N_a \end{aligned}$$

14 $$\begin{aligned} T_{total}= & \left( k_{b0}(P) + \frac{k_{b1}(P)}{N}\theta + \frac{ k_{b2}(P)}{N^2}\theta ^2\right) N_a \end{aligned}$$

This modeling approach provides a foundation for developing omnidirectional actuators. Such an actuator is realized by arranging multiple identical actuators in parallel and controlling them through separate pressure inputs, both in magnitude and timing. Equations 12-14 describe the behavior of the omnidirectional actuator when all constituent actuators are pressurized simultaneously.

## Application of Modeling Framework in Practical Scenarios

**Fig. 15:**
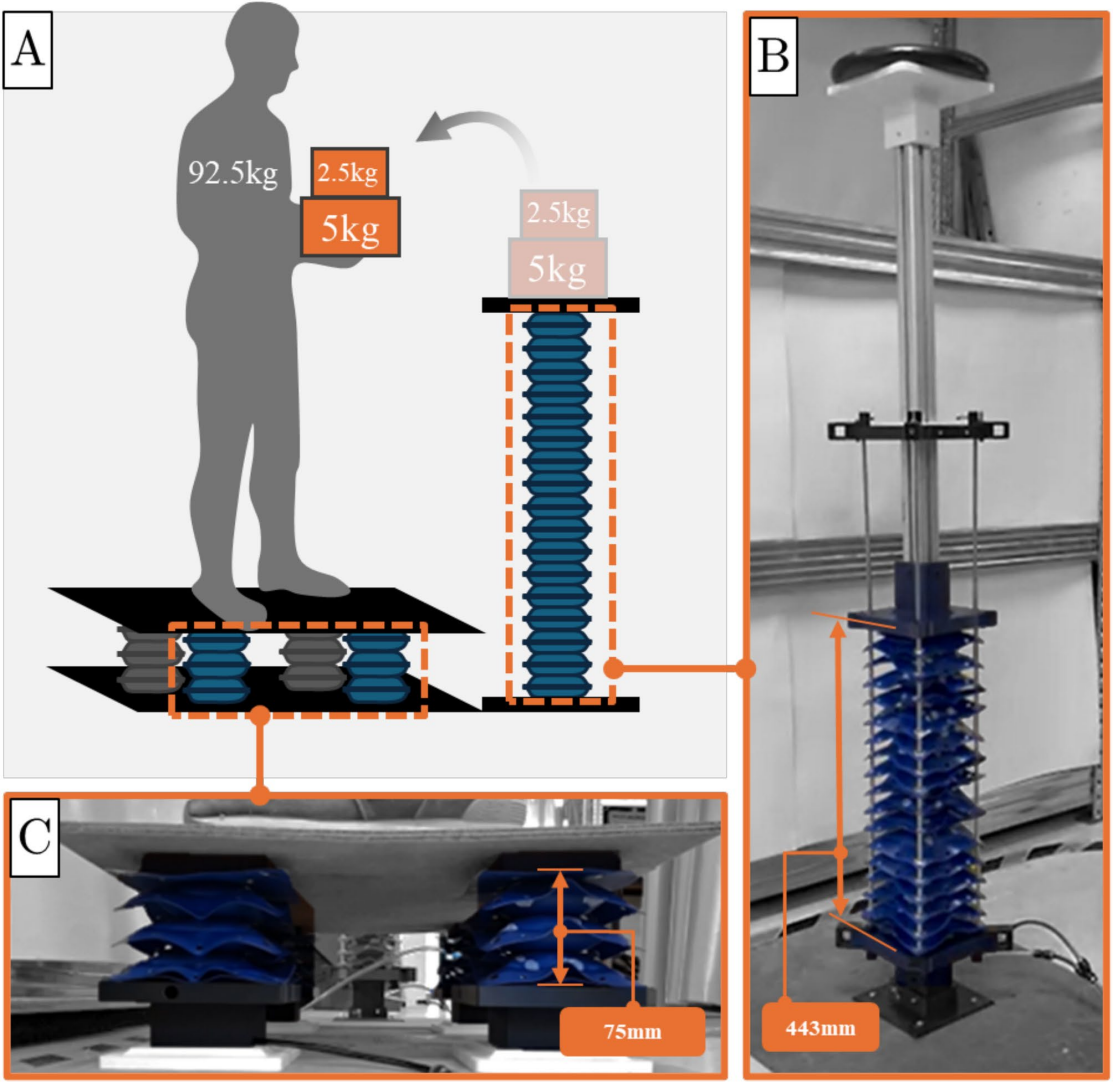
Application scenarios. (**A**) Illustration of two application scenarios: lifting a 7.5 kg weight to a height of 500 mm using a single elongating actuator with *N* chambers, and lifting a 92.5 kg human carrying an additional 7.5 kg to a height of 80 mm using $N_a$ identical elongating actuators, each composed of three chambers. The required values $N=17$ and $N_a = 4$ were determined using the spring model equations (Eq. 17) to achieve the target displacements. (**B**) Experimental validation of the 17-chamber actuator from the first application scenario, achieving a vertical lift of 443 mm at 0.05 MPa, corresponding to an 11% deviation from the predicted value. (**C**) Experimental setup of the second use case scenario, involving four three-chamber elongating actuators operating in parallel to lift a 100 kg total load to 80 mm at 0.1 MPa. The experimental lift reached 75 mm, showing a 6.6% deviation, thereby validating the model’s predictive accuracy in multi-actuator systems.

In this section, the application of the proposed spring-based modeling framework is demonstrated through two representative examples that address common challenges in manual handling and industrial assistance tasks. These case studies illustrate how the framework facilitates task- and requirement-specific design of soft inflatable actuators, thereby significantly streamlining the development process. The elongating actuator is chosen as the illustrative case due to its suitability for vertical lifting applications. As depicted in Fig. 15A, the first scenario involves lifting a relatively light load to a substantial height, while the second scenario focuses on lifting a much heavier load to a shorter vertical distance.

In the first scenario, the objective is to determine the number of chambers N required in a single elongating actuator to lift a 7.5 kg weight ($F_1 = 90 N$, including the structure’s weight) to a vertical height of $L_{target}=$ 500 mm when inflated at a pressure P = 0.05MPa. Given that the unloaded maximum elongation of the unit cell is ($L_{1}=44.5 mm$), the total no-load elongation length of an actuator with N chambers can be calculated with Eq. 15. When the load is applied, the actuator compresses to meet the target height, resulting in a displacement shown in Eq. 16:

15 $$\begin{aligned} L_N= & L_1\cdot N \end{aligned}$$

16 $$\begin{aligned} x= & L_N - L_{target} \end{aligned}$$

Using the spring model (Eq. 7) and substituting the known parameters from Eq. 15 and 16, the number of chambers N is determined by solving the second-order polynomial presented in Eq. 17.

17 $$\begin{aligned} FN^2 - k_{e1}(P)Nx - k_{e2}(P) x^2 = 0 \end{aligned}$$

The equation yields two solutions: $N_1=16.84$ and $N_2=5.18$. However, since the second solution does not meet the displacement requirement, it is discarded, leading to the design of an actuator equipped with 17 chambers.

When tested experimentally, the actuator achieved a vertical lift of 443 mm as shown in Fig. 15B. This result aligns with the previously observed plateau in modeling accuracy for elongating actuators, exhibiting an approximate 11$\%$ deviation, thereby confirming the reliability of the spring model for large values of N.

In the second scenario, the task is to lift a 92.5 kg human participant carrying an additional 7.5 kg load to a target height of 80 mm. The goal is to determine the number $N_a$ of three-chamber elongating actuators connected in parallel needed to achieve the required lift when inflated at a pressure of P = 0.1 MPa. The corresponding displacement x is derived from Eq. 16, using N = 3 chambers and a target height of $L_{target} =$80mm.

By solving Eq. 12 for $N_a$ and substituting the known parameters, the required number of actuators was calculated to be $N_a=3.87$. Rounding up, four actuators were selected for the design.

Experimental testing of the fabricated setup demonstrated a resulting lift of 75 mm as shown in Fig. 15C, corresponding to a 6.6$\%$ deviation from the desired value. The slightly higher error, compared to the 3.53$\%$ deviation observed for a single three-chamber actuator, is attributed to the increased complexity of the current experimental setup. When lifting heavy loads outside the controlled laboratory environment, additional sources of error, such as lateral bending of the actuators and deformation of the platform’s upper surface, can arise. Nonetheless, the observed deviations remain within acceptable limits, highlighting the strong predictive capability of the proposed framework.

Additional application scenarios that demonstrate the predictive capability of the proposed framework are presented in the Supplementary Materials (see Movie S2).

## Discussion and future directions

This study introduces, for the first time, a comprehensive taxonomy of soft inflatable fabric actuators centered around the stiffening actuator as the fundamental unit cell. Within this framework, a variety of actuator types – such as elongating, contracting, bending, twisting, and omnidirectional – can emerge either from spatial configurations of the basic unit cell (i.e., combining multiple units in series or parallel) or from structural modifications (on-body configurations) of the unit cell itself. The unit cell actuator was chosen as the foundational element due to its core mechanical behavior, which can be effectively modeled using spring analogies. These spring models provide an extensible foundation for predicting the behavior of more complex actuator types.

To model the nonlinear mechanical response of the actuators in a simple yet reliable way, polynomial regression was employed. Polynomials offer a flexible functional form that can approximate complex behaviors while remaining both computationally efficient and analytically straightforward. An additional motivation for this choice was the desire to preserve an analogy with the classical spring law, thereby maintaining conceptual continuity with conventional spring models. The polynomial order for each actuator type was determined by balancing accuracy against computational efficiency: regression analyses were performed with progressively higher orders, and the lowest-order fit that achieved satisfactory error metrics was selected. For elongating and bending actuators, quadratic polynomials were sufficient to capture the dominant nonlinearities without overfitting, whereas contracting actuators required higher-order terms to represent their stronger nonlinear trends. This systematic approach ensures accurate predictions while preserving the generality and applicability of the framework across different actuator designs.

By representing the unit cell actuator as a compression/extension or torsional spring, the performance of advanced actuators can be approximated through in-series and parallel combinations of these spring elements. FEM simulations and experimental validations confirmed that this modeling approach accurately predicts the performance of elongating, contracting, and bending actuators across different chamber counts and inflation pressures. Inflation pressure was found to have a direct, linear correlation with spring stiffness – higher pressures produce greater stiffness consistently across all actuator types. For elongating actuators, prediction error tends to increase with the number of chambers but plateaus around 10–11$\%$. This residual error can be attributed to structural instabilities that arise during operation. In particular, once the number of chambers exceeds five, the actuators tend to deviate from purely vertical extension and exhibit lateral bending upon inflation. This introduces discrepancies between the idealized model, which assumes uniform elongation, and the actual experimental response. Although lateral deformation was constrained in both simulations and physical tests to isolate vertical motion, residual tendencies toward bending still contribute to the higher prediction error in multi-chamber configurations. Importantly, this effect stabilizes at approximately 10–11$\%$, suggesting that while additional chambers amplify bending tendencies, the dominant sources of error become saturated, and the model continues to capture the primary elongation mechanics with consistent accuracy. In contrast, contracting and bending actuators exhibit relatively constant predictive accuracy as the number of chambers increases, with deviations remaining similar across larger configurations. For contracting actuators, the proposed framework yields an error of approximately 11$\%$, independent of the number of chambers. For bending actuators, the error ranges between 2$\%$ and 4$\%$, with a slight decrease observed for configurations with more chambers. This decrease is attributed to the comparison being performed at higher torque values, due to the experimental limitations discussed earlier.

In the context of soft robotic actuator design, errors of this magnitude are considered acceptable, as they fall within the range typically reported for empirical or reduced-order models. This level of accuracy is sufficient to guide the design and optimization process without resorting to computationally expensive full-scale FEM simulations. Moreover, the framework offers rapid scalability across actuator geometries, making it a practical and efficient design tool despite the modest residual error.

**Fig. 16:**
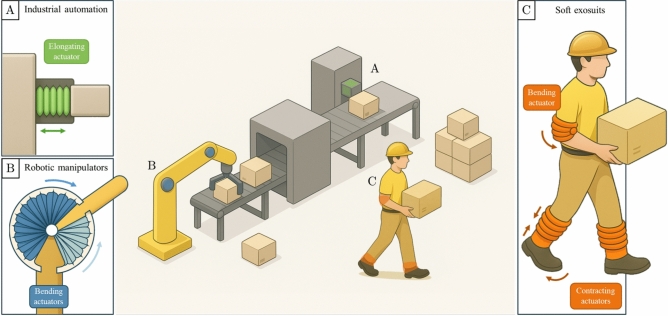
Conceptual application scenarios for soft inflatable fabric actuators. (**A**) Elongating actuators in industrial automation, where their lightweight structure and high force-to-weight ratio enable safe and efficient handling of loads. (**B**) Bending actuators in robotic manipulators, providing compliant and adaptive motion for grasping and manipulation. (**C**) Contracting and bending actuators in wearable exosuits, assisting human movement by supporting lifting, posture stabilization, and locomotion.

The versatility of soft inflatable fabric actuators spans a broad range of real-world applications, as indicatively illustrated in Figure 16. In industrial automation (Figure 16.A), elongating actuators can serve as lightweight, compliant replacements for rigid linear actuators, offering not only safe interaction with delicate components but also a high force-to-weight ratio that is advantageous in repetitive production tasks. In robotic manipulators (Fig. 16.B), bending actuators can be integrated into joints to provide adaptive and compliant motion. Finally, in wearable soft exosuits (Figure 16.C), both contracting and bending actuators can be employed to support human movement, assisting with lifting, posture stabilization, and locomotion while reducing the physical strain of load-bearing tasks. Beyond the applications highlighted in the figure, these actuators have also been demonstrated in other domains discussed in the introduction, including biomedical devices (e.g., endoscopic tools, tissue manipulation, and artificial heart systems) and soft grippers for delicate object handling. Such breadth further emphasizes their adaptability across fields where lightweight, compliant, and high-performance actuation is critical. The spring-based framework developed in this work enables these diverse applications by providing a unified, computationally efficient method for predicting actuator behavior, thereby facilitating the systematic design and scaling of actuators to meet specific functional requirements.

The practical utility of the proposed taxonomy and spring-based modeling framework was demonstrated through two representative use cases, each addressing different actuation demands. The first scenario involved designing an elongating actuator to lift a relatively light load over a large displacement, where the required number of chambers was calculated via the spring models, eliminating the need for iterative prototyping or extensive simulations. The second scenario focused on lifting a heavier load over a shorter distance by employing multiple actuators in parallel, with the framework determining the optimal number of actuators needed. These examples highlight the framework’s capability to tailor actuator design to specific task requirements, whether maximizing displacement for low-load applications or maximizing force for heavy loads. Notably, both scenarios confirmed that the behavior of multi-chamber actuators can be accurately predicted by extrapolating from a single high-fidelity FEM simulation of the fundamental unit cell, significantly reducing design time and computational costs.

Additionally, the overall mechanical behavior of multi-chamber actuators can be finely tuned by adjusting the inflation pressure of the individual chambers. Each chamber acts as a spring whose stiffness is pressure-dependent; thus, modifying internal pressures effectively controls the actuator’s global compliance and deformation characteristics. This pressure-based tuning offers a versatile, programmable approach to stiffness control without mechanical redesign, enhancing the adaptability and responsiveness of soft actuator systems in dynamic environments.

The actuators examined in this study were constructed with fixed material properties and geometry. When multiple actuators with identical unit cell characteristics are arranged in series or parallel, their multi-chamber behavior can be reliably predicted using the proposed framework. In contrast, variations in material properties or unit cell dimensions modify the actuator’s response, leading to changes in stiffness as well as shifts in the maximum achievable force or torque. These effects can still be accommodated within the spring-based models by recalculating the stiffness coefficients while retaining the same fundamental spring equation for each actuator type. For multi-chamber actuators composed of unit cells with different materials or geometries, the framework remains applicable, but the springs will exhibit different stiffness values, requiring slight modifications to the equations to account for the heterogeneous configuration. To address such cases, the same methodology outlined in this work can be applied, with springs of different stiffness algebraically combined. Formalizing and experimentally validating these more complex, heterogeneous actuator configurations would be of great interest for future investigation.

Generality was a central consideration in developing the framework, which was validated across a wide range of inflation pressures (0.05–0.2 MPa) and chamber counts (with up to 10 chambers being characterized experimentally, and a 17-chamber actuator tested as a demonstrator). Deviations observed in the larger actuators remained comparable to those in smaller configurations, underscoring the robustness and scalability of the approach beyond the explicitly tested ranges. To further enhance generality and scalability, the force–displacement relationships can also be expressed in dimensionless form. Dimensionless plots remove dependence on absolute material and geometric parameters, enabling actuators of different sizes or materials to be directly compared on a common basis. This approach facilitates scalability, allowing predictions across diverse actuator designs without the need for repeated experimental or numerical characterization. A detailed description of the methodology is provided in our earlier work^53^, where the governing dimensionless ratios were derived and validated. The dimensionless force–displacement curves for the present actuators are provided in Supplementary Materials, Section 8, illustrating how data from multiple inflation pressures collapse onto a unified curve that highlights the underlying mechanics independent of scale. It should be noted, however, that dimensional analysis does not imply unlimited scalability: at extreme scales, whether very large, where factors such as self-weight and structural stability become dominant, or very small, where surface effects prevail, the assumptions underlying the model would no longer hold, and additional considerations would be required.

In developing simplified models of soft inflatable actuators, certain assumptions are inevitably introduced to enable tractable analysis and comparison with experiments. At the experimental level, frictional effects and possible non-uniformities in pressure distribution are neglected. Within the finite element simulations, material phenomena such as viscoelasticity and hysteresis are not accounted for. In addition, the spring-like analytical formulation further reduces complexity by assuming actuator displacement to be independent of the applied pressure. These simplifications allow for systematic evaluation of actuator performance but also introduce limitations. For instance, while polynomial spring-based models provide a general framework for describing actuators with diverse geometries and materials, the resulting stiffness coefficients are geometry- and material-dependent, and thus must be determined specifically for each actuator configuration. Furthermore, the studied pressure is limited to 0.05–0.2 MPa range. Higher pressure could be achievable with more sophisticated heat-sealing techniques (ultrasonic or radio frequency welding). Moreover, although an explicit dynamic solver is used in the finite element analysis to accurately capture the high non-linearities, the actuator force/torque output is accurately predicted in static or quasi-static conditions, restricting its applicability in time-varying conditions. Finally, the lack of cyclic testing prevents assessment of fatigue and long-term durability, aspects that will be addressed in future work.

A promising direction for future work is extending the current framework beyond static analysis to capture the dynamic behavior of soft inflatable fabric actuators using a spring-model approach. In this context, the time-dependent responses of these complex systems can be modeled through active spring elements in the analytical dynamics equations, with stiffness treated as a function of inflation pressure. Integrating this dynamic, spring-based modeling into model-based control algorithms would enable efficient, adaptive, and precise control strategies. Embedding the spring models within real-time control schemes would allow these strategies to account for the rapid dynamic phenomena characteristic of soft actuators.

Future research will also investigate new on-body configuration designs, focusing on how structural reinforcements and material constraints influence the stiffness characteristics captured by the proposed spring models. Additionally, efforts will be directed toward modeling and characterizing omnidirectional actuators when their constituent actuators are pressurized independently to achieve more complex motions, utilizing the developed spring-based framework. These studies aim to further validate and expand the applicability of the proposed taxonomy and modeling approach, thereby reinforcing its potential as a generalizable and scalable tool for the design, analysis, and control of soft inflatable fabric actuators.

## Conclusions

This study introduces a unified taxonomy and spring-based modeling framework for soft inflatable fabric actuators, centered on the stiffening actuator as a fundamental unit cell. By modeling this core unit as a nonlinear spring and validating its mechanical behavior through both FEM simulations and experimental testing, we demonstrated that the performance of more complex actuators, such as elongating, contracting, and bending actuators, can be reliably predicted from the response of a single unit. The framework effectively models multi-chamber actuators as systems of springs connected in series or parallel, with force and torque outputs expressed as functions of the number of chambers and the inflation pressure. Because each actuator module is modeled independently and assembled through analytical spring combinations, the framework scales effectively to high-degree-of-freedom systems, enabling the design of full robotic limbs or distributed actuation arrays without retraining or re-simulation.

Two representative application scenarios further illustrated the practical utility of the framework, enabling rapid and task-specific actuator synthesis without reliance on iterative physical prototyping or computationally intensive simulations. By significantly reducing design complexity and development time, this modeling approach offers a scalable, generalizable, and efficient tool for the design and deployment of soft robotic systems tailored to specific performance requirements.

## Supplementary Information

Supplementary Information 1.

Supplementary Information 2.

Supplementary Information 3.

Supplementary Information 4.

## Data Availability

All data generated or analysed during this study are included in this published article and its supplementary information files.
