# Supplementary material for: A unified framework for soft inflatable fabric actuators
Source: Sci Rep. 2025 Nov 24;15:41629. doi: 10.1038/s41598-025-25643-8 (PMC12644749; doi:10.1038/s41598-025-25643-8)
Supplement: Supplementary file 4 — Supplementary Information 4. [file 41598_2025_25643_MOESM4_ESM.pdf]

Supporting Information

A Unified Taxonomy for Soft Inflatable Fabric Actuators

*Odysseas Simatos, Konstantina Tsintzira, Grigorios Chatziathanasiou, and Panagiotis Polygerinos**

1. Uniaxial test and final orthotropic material properties

   The material used for fabrication prototypes is a double-sided TPU-coated Nylon (840D). To accurately ascertain the mechanical properties of the material, a series of uniaxial tests were conducted in accordance with the ISO 527-1:2019 guidelines, such that the FEM model is accurately structured. These tests were performed using a universal testing machine (CTM6005, CTM, China) and its clip-on extensometer, as illustrated in the S1 figure.

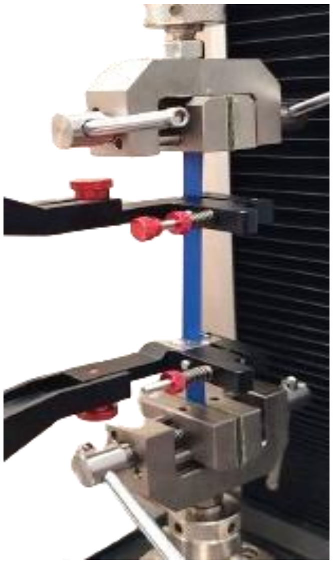

Figure S1: Uniaxial test

The tests were conducted for the warp, weft and bias direction, to find the Young's Moduli in the respective directions (E_0=361.4 MPa, E_{90}=183.3 MPa, and E_{45}=116.7MPa). Each experiment was conducted five times and subsequently the mean values were calculated. The in-plane Poisson ratio was chosen as the typical one in fabric TPU materials (v_{12}=0.35), and the in-plane shear modulus (G_{12}) was approximated by the equation below, based on the modulus obtained from the 45° test.

$$G_{12} = \frac{E_{45^o}}{2(1+v_{12})} = 43.2 \text{ MPa}$$

For the out-of-plane properties, small nonzero values were used.

2. Fabrication Process

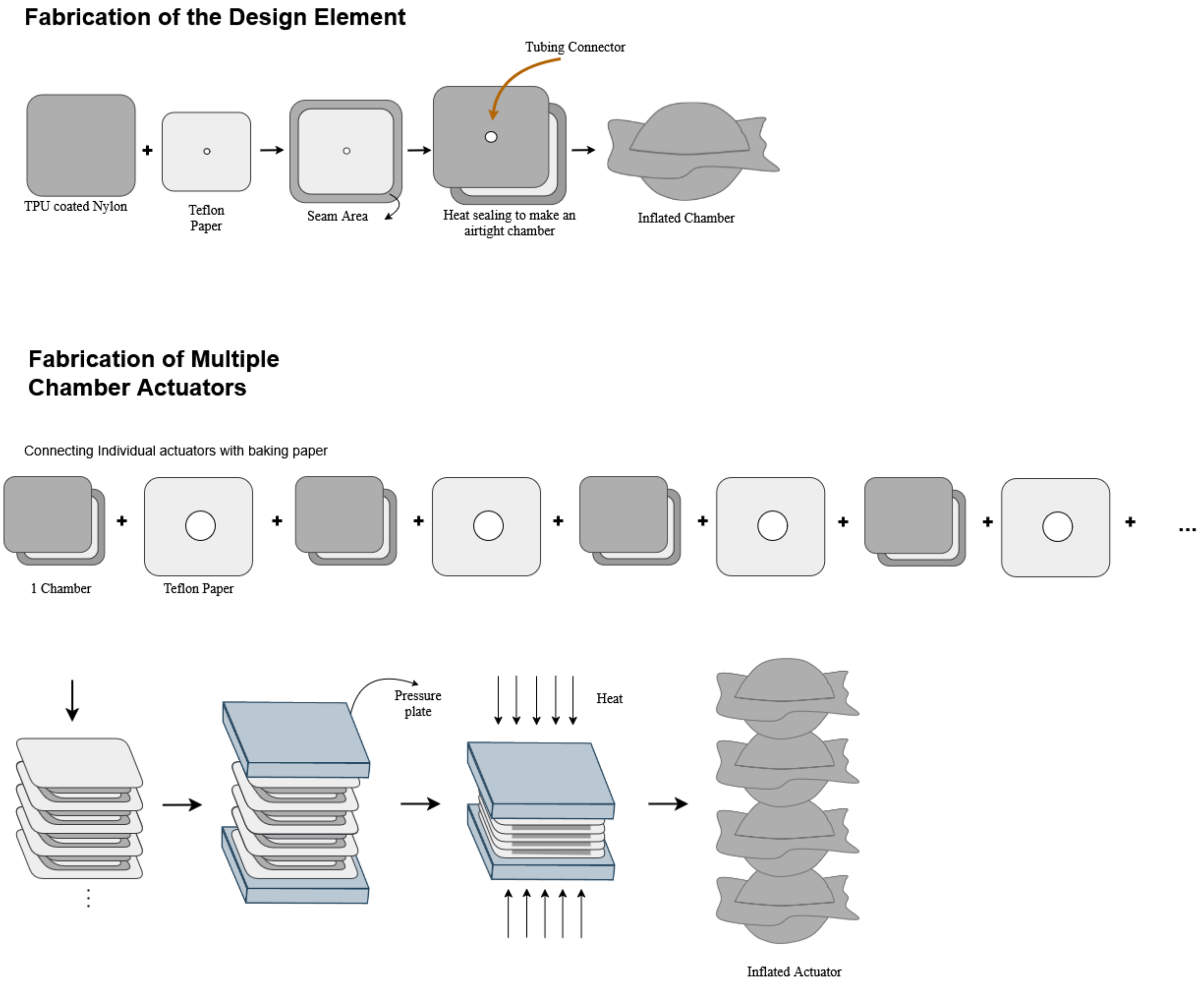

Figure S2: Fabrication process of a) design element b) multi-chamber actuator

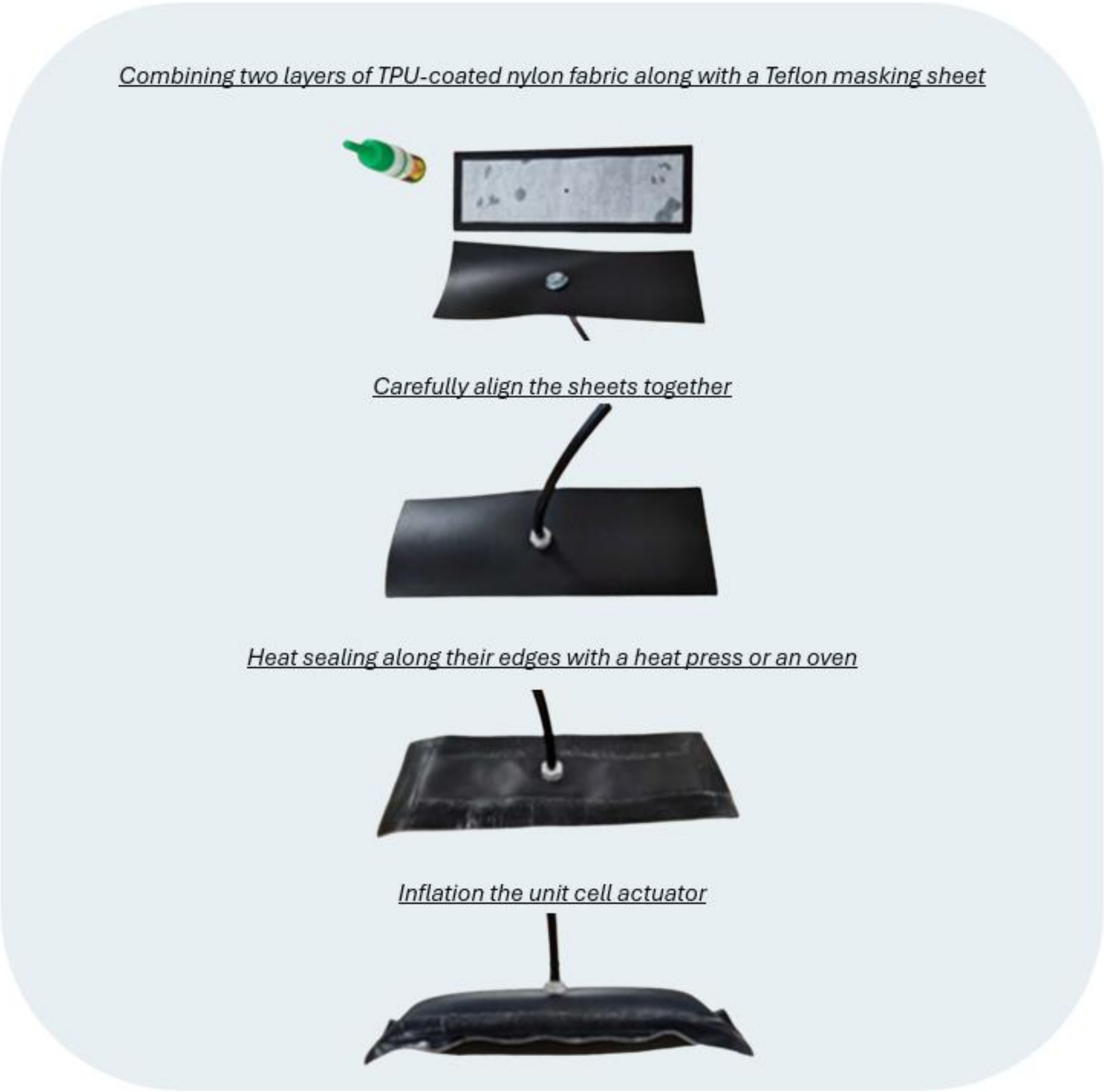

Figure S3: Fabrication of a physical prototype of a unit cell actuator

3. <u>Selected Designs for each type of actuator</u>
   The mechanical designs and dimensions of each chamber used for the elongating, contracting, and bending actuators are depicted below. All dimensions are in millimeters.

- Elongating actuator

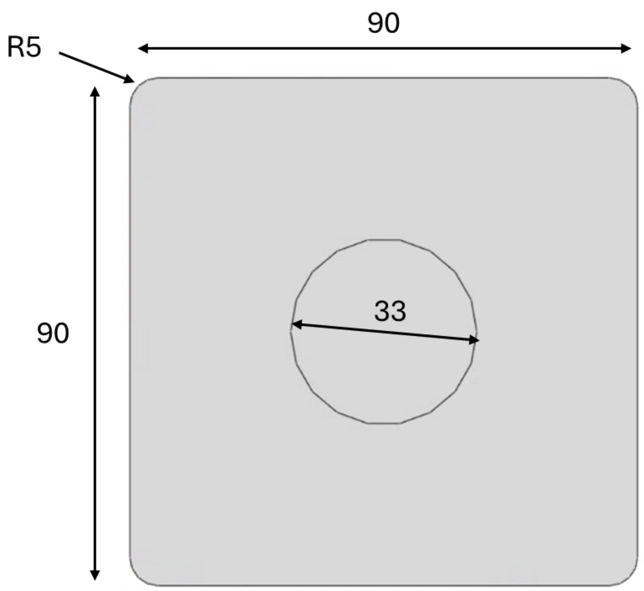

Figure S4: Design of the elongating actuator's chamber.

- Contracting actuator

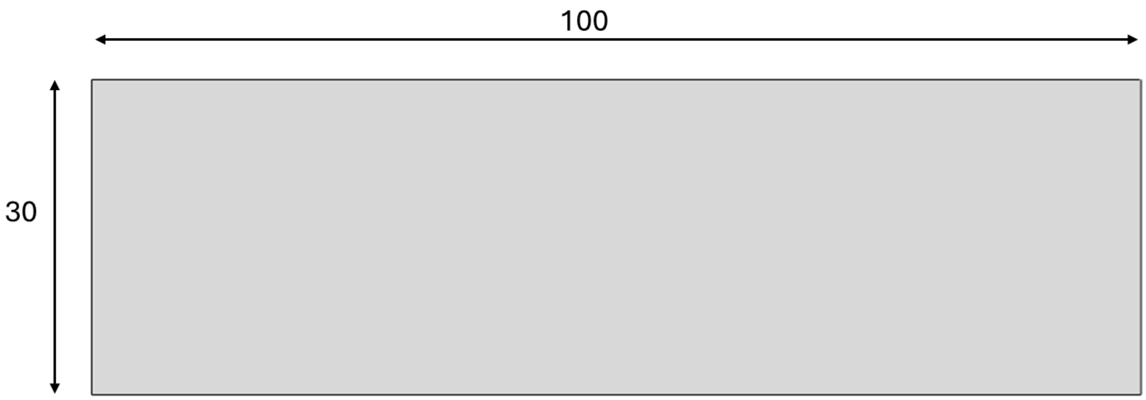

Figure S5: Design of the contracting actuator's chamber

- Bending actuator

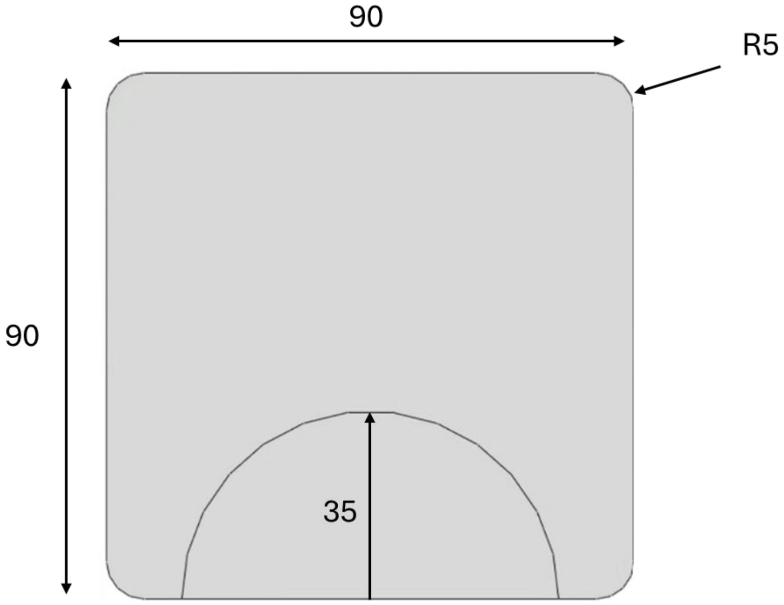

Figure S6: Design of the bending actuator's chamber

4. <u>Physical prototypes of multi-chamber actuators</u>

- Elongating

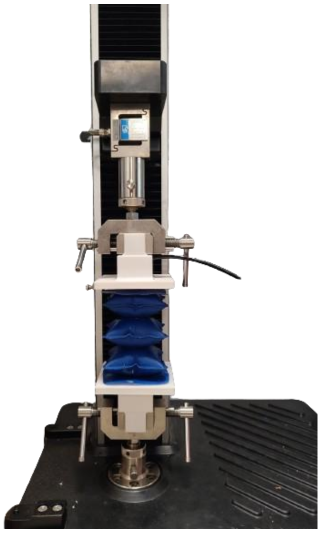

Figure S7: Three chambers elongating

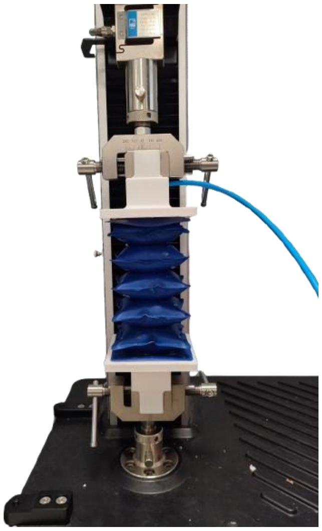

Figure S8: Five chambers elongating

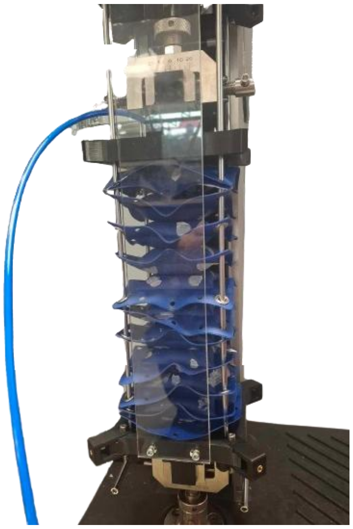

Figure S9: Ten chambers elongating

- Contracting

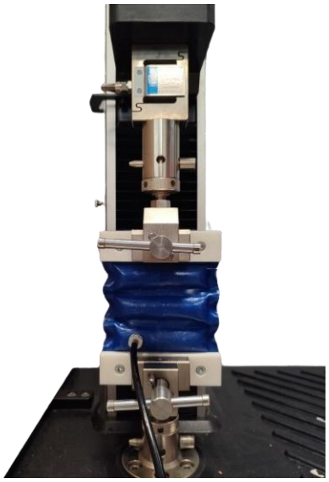

Figure S10: Three chambers contracting

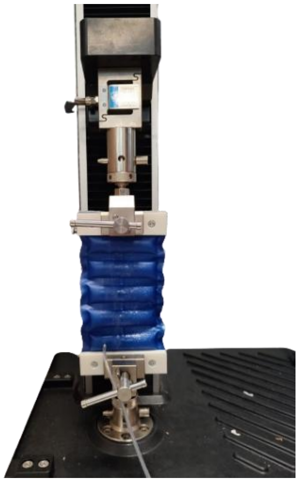

Figure S11: Five chambers contracting

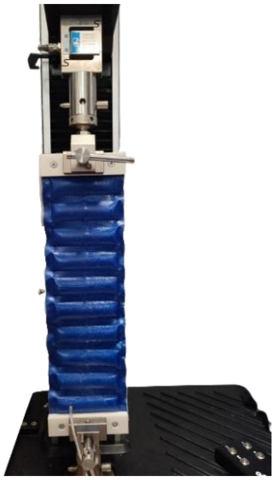

Figure S12: Ten chambers contracting

- Bending

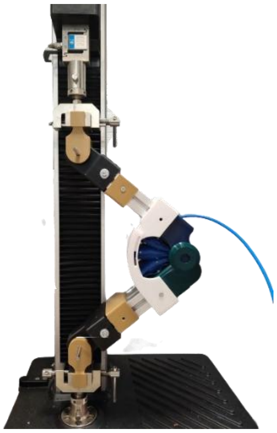

Figure S13: Three chambers bending

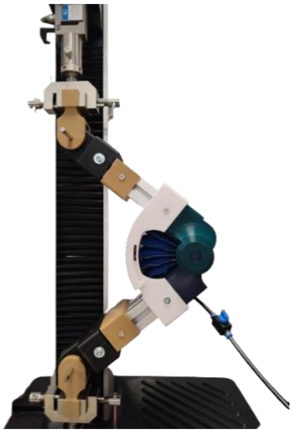

Figure S14: Five chambers bending

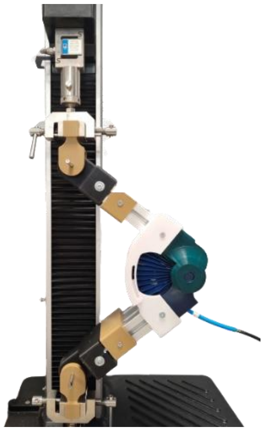

Figure S15: Ten chambers bending

5. <u>Torque calculation</u>

The force output F_M is measured using the load cell. The corresponding actuator force F_A and actuator torque T are calculated using Equations (S1) and (S2), based on the measurable distances d_A and d_M between the joint center and the points where F_A and F_M are applied.

$$F_A = F_M \cos(\theta/2)\, d_M/d_A \tag{S1}$$

$$T = F_A d_A \tag{S2}$$

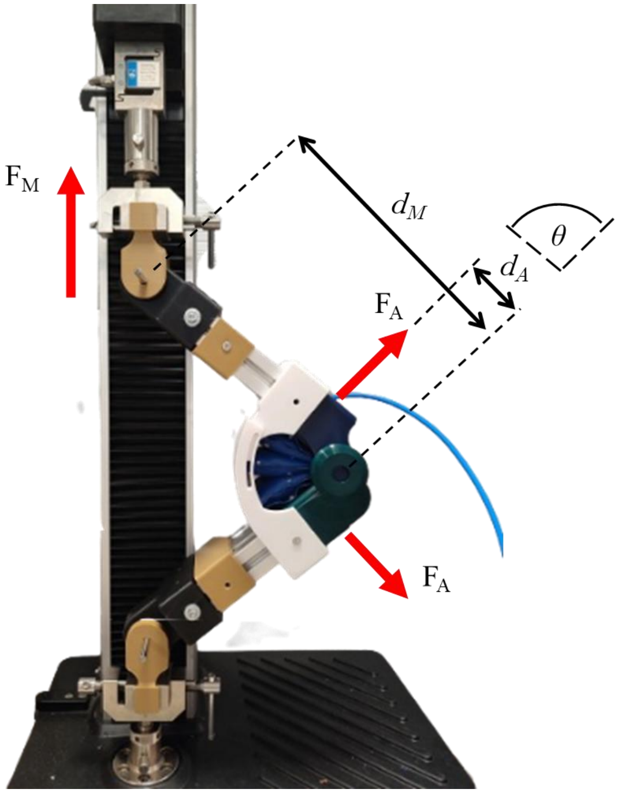

Figure S16: The experimental setup of the bending actuator, including the artificial joint, and the UTM.

6. <u>Mesh convergence analysis</u>

A mesh convergence analysis was conducted for each actuator type to achieve an optimal trade-off between computational cost and accuracy. Various mesh resolutions were tested, and convergence was established when additional refinement produced only minimal variations in the simulated force or torque output.

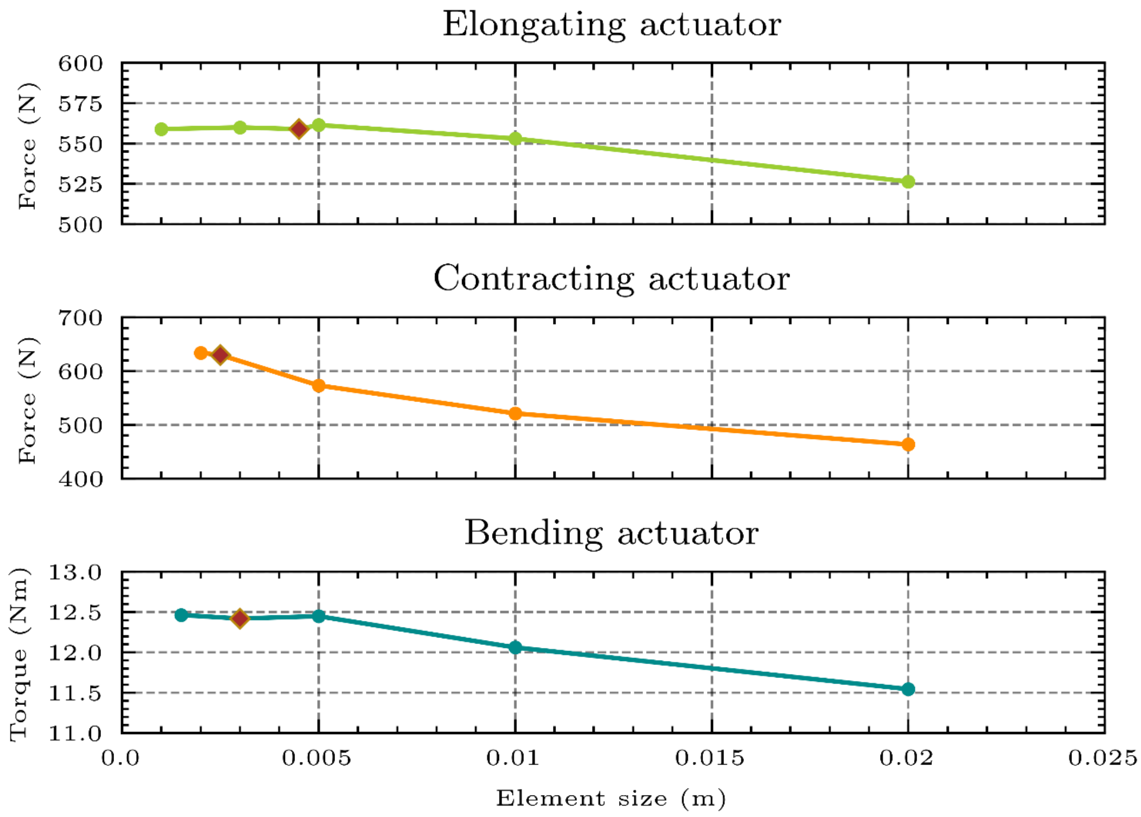

Figure S17: The mesh convergence study for the elongating, contracting, and bending actuator. The red diamond indicates the selected element size after convergence was achieved.

7. <u>Experiments</u>

To verify the consistency of the experimental procedure, each mechanical test of the unit cell was repeated seven times. The mean values and standard deviations of the collected data are presented in Figures S18–S20. The experiments demonstrate excellent reproducibility, with standard deviations consistently below 5% of the mean value, in accordance with commonly accepted guidelines for reliable measurements. The raw data from all experiments are provided in the Raw_data.xlsx file.

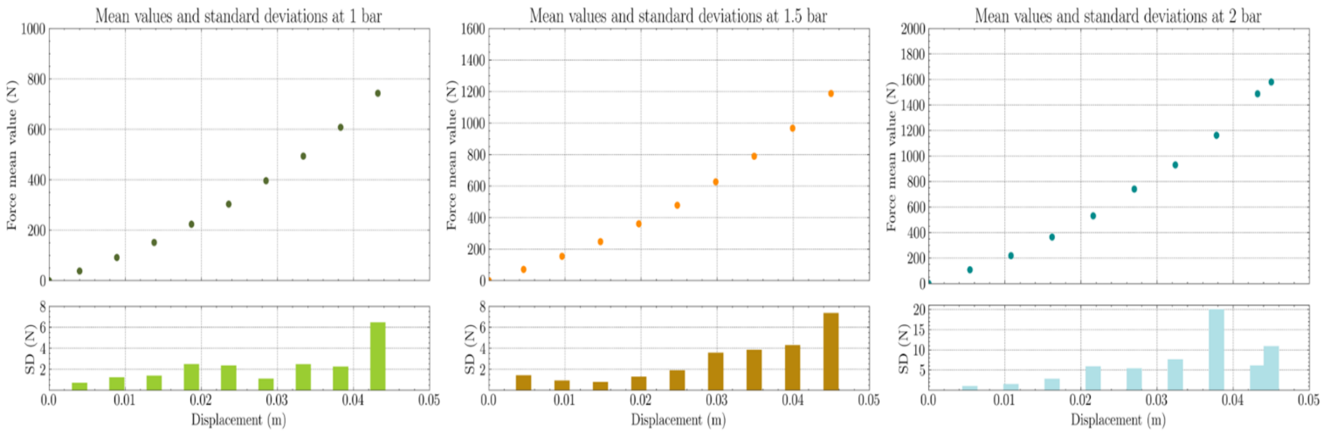

Figure S18: Mean values and standard deviations obtained from seven repeated compression tests of the unit cell for three pressure levels.

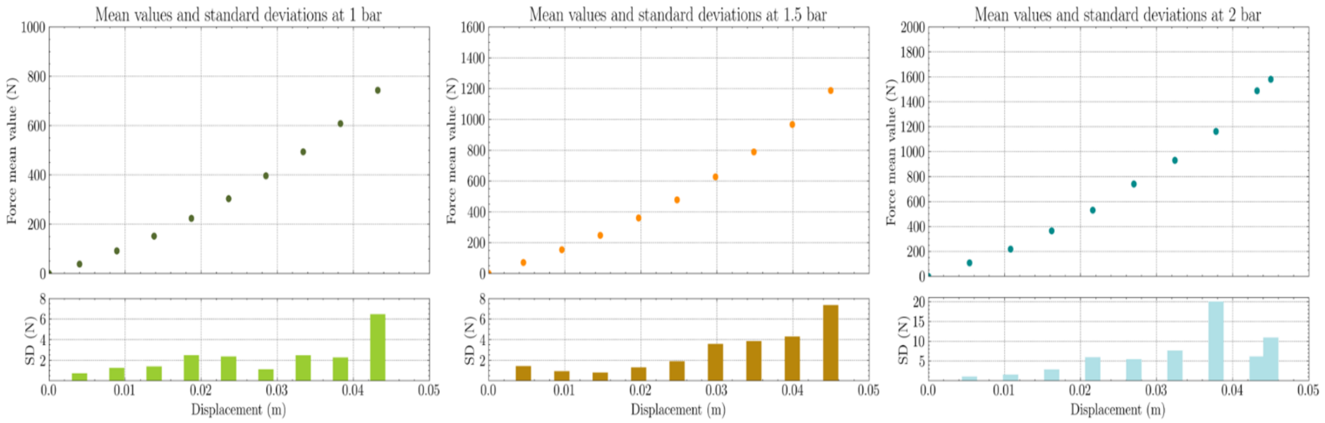

Figure S19: Mean values and standard deviations obtained from seven repeated tensile tests of the unit cell for three pressure levels.

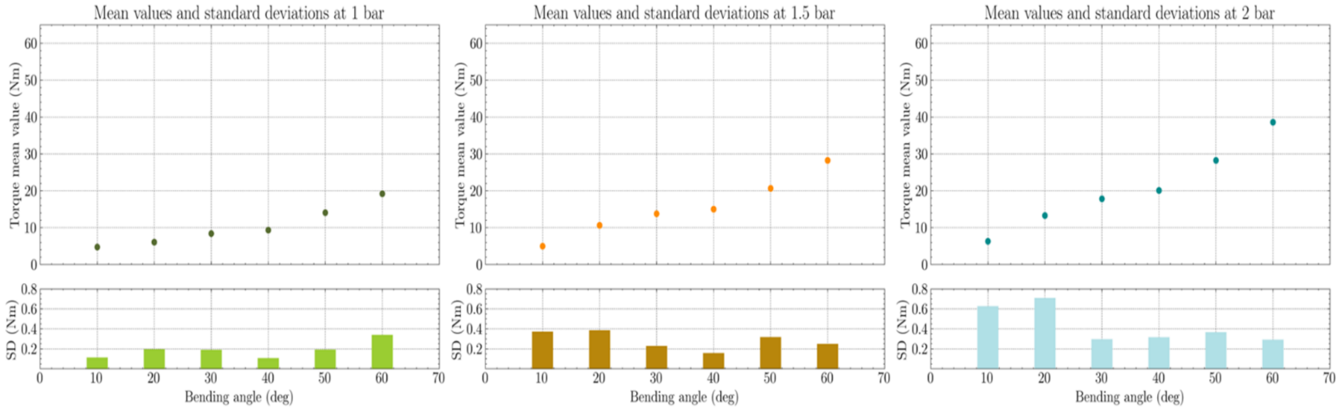

Figure S20: Mean values and standard deviations obtained from seven repeated bending tests of the unit cell for three pressure levels.

8. Dimensionless relationships

To further broaden the generality of this study and demonstrate the integration of dimensional analysis within the proposed spring-based framework, the force–displacement and torque–angle relationships were reformulated in dimensionless form. Such representations are particularly powerful as they eliminate dependence on specific geometric and material parameters, thereby enabling direct comparison of actuator performance across different sizes, materials, and operating conditions. This approach inherently facilitates scalability, allowing predictions to extend across a wide design space without the need for repeated experimentation or simulation for each new configuration.

Achieving complete generalization, in which force–displacement curves fully characterize each actuator type across all possible dimensions, materials, and pressures, would require the comprehensive methodology presented in our earlier work [33], involving the construction of multi-variable dimensionless maps. While this level of generalization lies beyond the scope of the present study, here we focus on providing representative examples that illustrate the utility of dimensional analysis when applied to the spring-based modeling framework.

Accordingly, Figures S21–S26 present the dimensionless plots corresponding to the actuators analyzed in the main text, demonstrating how data obtained under different inflation pressures collapse onto unified curves defined by their governing dimensionless parameters.

The dimensionless stiffness coefficients are obtained using the same methodology described in the main manuscript, with force and displacement expressed in their dimensionless forms. Notably, the spring-based equations introduced in the manuscript remain directly applicable in this dimensionless framework without modification.

A

Name	Variable	Type
Dimensionless force	ϕ	$F/(Pl^2)$
Dimensionless displacement	χ	d/l
Geometrical ratio	λ	w/l
Number of chambers	n	inherently dimensionless
Dimensionless thickness	β	b/l
Dimensionless elastic modulus	ϵ	E/P
Poisson's ratio	ν	inherently dimensionless

B

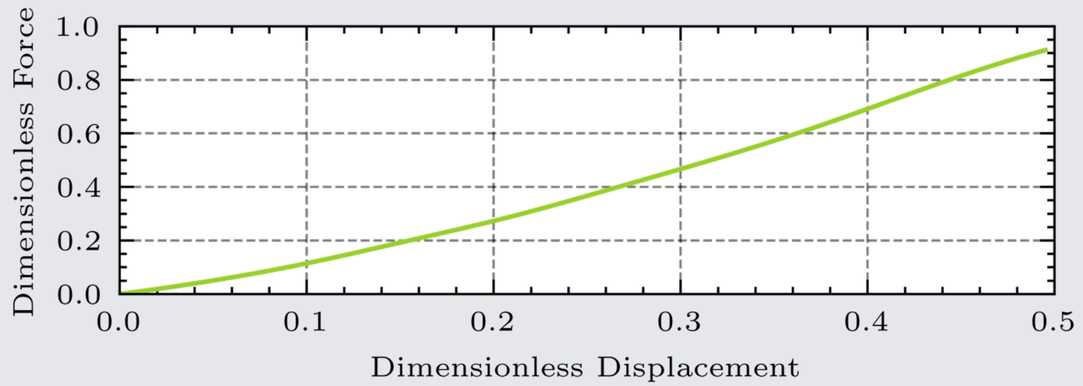

Figure S21: (A) Dimensionless variables used for the compression test of the unit cell actuator. (B) Dimensionless force–displacement curve of the compression test, demonstrating the convergence of the three curves from the main manuscript into a single unified response.

A

Name	Variable	Type
Dimensionless force	ϕ	$F/(Pl^2)$
Dimensionless displacement	χ	d/l
Geometrical ratio	λ	w/l
Number of chambers	n	inherently dimensionless
Dimensionless thickness	β	b/l
Dimensionless elastic modulus	ϵ	E/P
Poisson's ratio	ν	inherently dimensionless

B

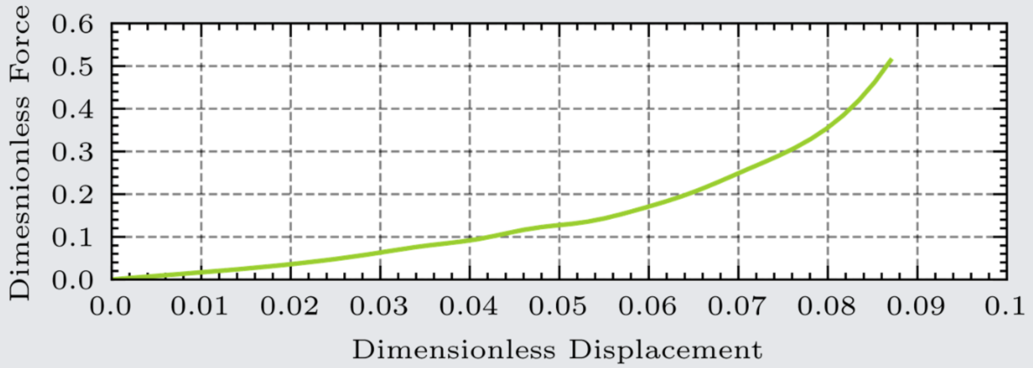

Figure S22: (A) Dimensionless variables used for the tensile test of the unit cell actuator. (B) Dimensionless force–displacement curve of the tensile test, demonstrating the convergence of the three curves from the main manuscript into a single response.

A

Name	Variable	Type
Dimensionless torque	τ	$T/(Pl^3)$
Bending angle	θ	inherently dimensionless
Geometrical ratio	λ	w/l
Number of chambers	n	inherently dimensionless
Dimensionless thickness	β	b/l
Dimensionless elastic modulus	ϵ	E/P
Poisson's ratio	ν	inherently dimensionless

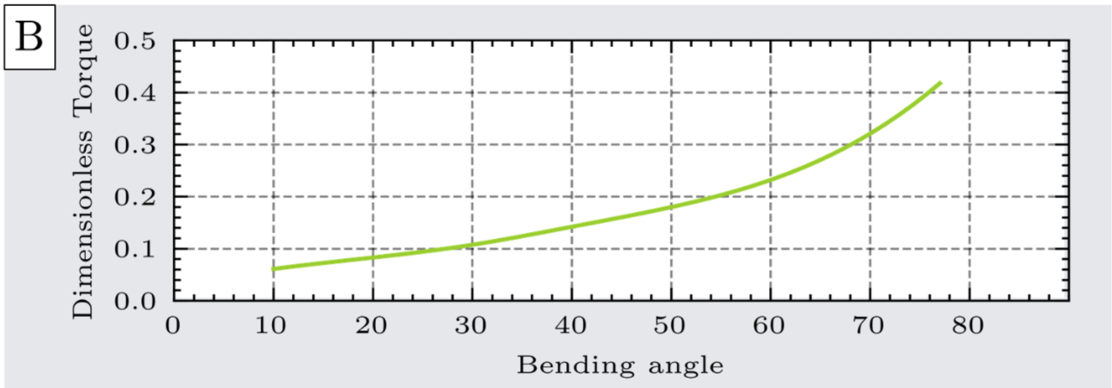

Figure S23: (A) Dimensionless variables used for the bending test of the unit cell actuator. (B) Dimensionless torque–angle curve of the bending test, demonstrating the convergence of the three curves from the main manuscript into a single response.

A

Name	Variable	Type
Dimensionless force	ϕ	$F/(Pl^2)$
Dimensionless displacement	χ	d/l
Geometrical ratio	λ	w/l
Number of chambers	n	inherently dimensionless
Dimensionless thickness	β	b/l
Dimensionless elastic modulus	ϵ	E/P
Poisson's ratio	ν	inherently dimensionless

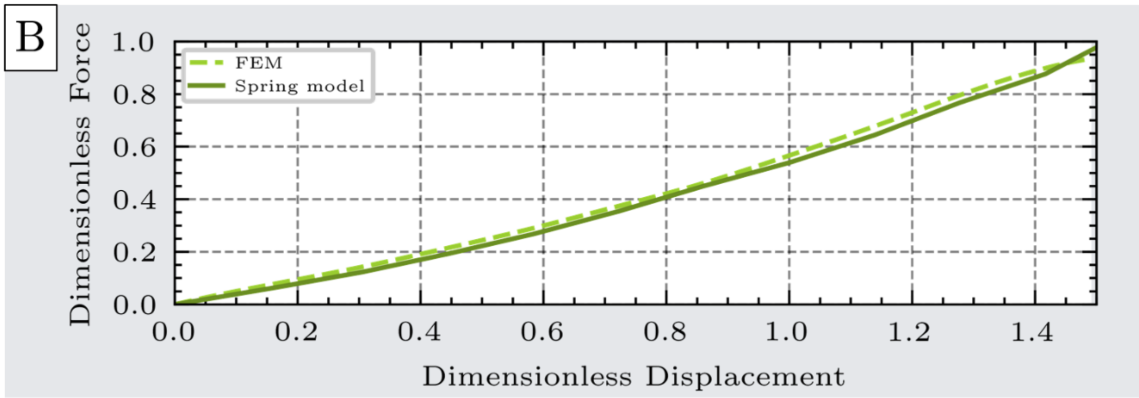

Figure S24: (A) Dimensionless variables defined for the elongating actuator. (B) Dimensionless force–displacement curves obtained from FEM simulations and the spring-based model, showing strong agreement between the two approaches and the collapse of data from different pressure levels onto a single unified curve.

A

Name	Variable	Type
Dimensionless force	ϕ	$F/(Pl^2)$
Dimensionless displacement	χ	d/l
Geometrical ratio	λ	w/l
Number of chambers	n	inherently dimensionless
Dimensionless thickness	β	b/l
Dimensionless elastic modulus	ϵ	E/P
Poisson's ratio	ν	inherently dimensionless

B

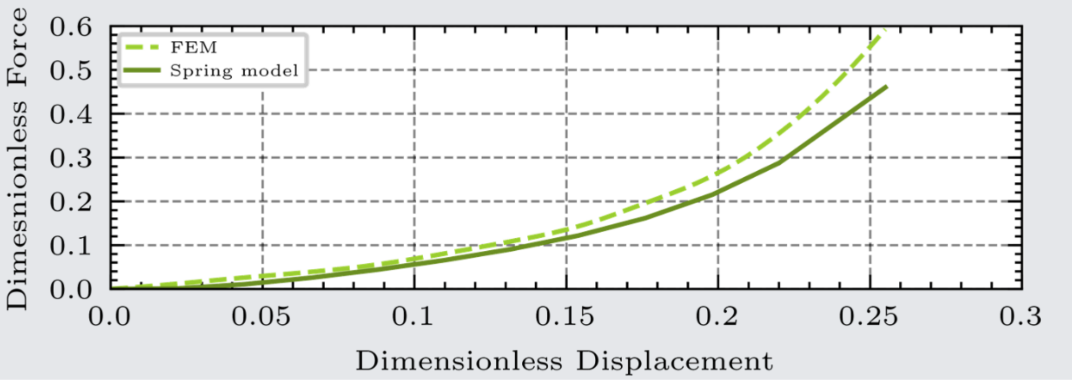

Figure S25: (A) Dimensionless variables of the contracting actuator. (B) Dimensionless force–displacement curves obtained from FEM simulations and the spring-based model, showing strong agreement between the two approaches and the collapse of data from different pressure levels onto a single unified curve.

A

Name	Variable	Type
Dimensionless torque	τ	$T/(Pl^3)$
Bending angle	θ	inherently dimensionless
Geometrical ratio	λ	w/l
Number of chambers	n	inherently dimensionless
Dimensionless thickness	β	b/l
Dimensionless elastic modulus	ϵ	E/P
Poisson's ratio	ν	inherently dimensionless

B

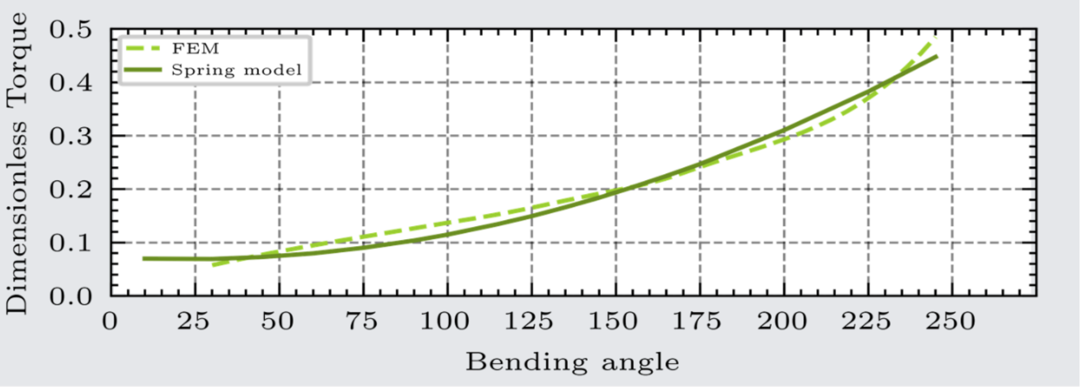

Figure S26: (A) Dimensionless variables of the bending actuator. (B) Dimensionless torque–angle curves obtained from FEM simulations and the spring-based model, showing strong agreement between the two approaches and the collapse of data from different pressure levels onto a single unified curve.
